# Impact of Fusarium-Derived Mycoestrogens on Female Reproduction: A Systematic Review

**DOI:** 10.3390/toxins13060373

**Published:** 2021-05-24

**Authors:** Carolyn W. Kinkade, Zorimar Rivera-Núñez, Ludwik Gorcyzca, Lauren M. Aleksunes, Emily S. Barrett

**Affiliations:** 1 Joint Graduate Program in Exposure Science, Department of Environmental Sciences, Rutgers University, Piscataway, NJ 08854, USA; 2 Environmental and Occupational Health Sciences Institute, Rutgers University, Piscataway, NJ 08854, USA; zr69@eohsi.rutgers.edu (Z.R.-N.); aleksunes@eohsi.rutgers.edu (L.M.A.); 3 Department of Biostatistics and Epidemiology, School of Public Health, Rutgers University, Piscataway, NJ 08854, USA; 4 Joint Graduate Program in Toxicology, Rutgers University, Piscataway, NJ 08554, USA; ljg96@gsbs.rutgers.edu; 5 Department of Pharmacology and Toxicology, Ernest Mario School of Pharmacy, Rutgers University, Piscataway, NJ 08854, USA; 6 Rutgers Center for Lipid Research, New Jersey Institute for Food, Nutrition, and Health, Rutgers University, New Brunswick, NJ 08901, USA

**Keywords:** mycotoxin, mycoestrogen, zearalenone, zeranol, pregnancy, fertility, female reproduction

## Abstract

Contamination of the world’s food supply and animal feed with mycotoxins is a growing concern as global temperatures rise and promote the growth of fungus. Zearalenone (ZEN), an estrogenic mycotoxin produced by *Fusarium* fungi, is a common contaminant of cereal grains and has also been detected at lower levels in meat, milk, and spices. ZEN’s synthetic derivative, zeranol, is used as a growth promoter in United States (US) and Canadian beef production. Experimental research suggests that ZEN and zeranol disrupt the endocrine and reproductive systems, leading to infertility, polycystic ovarian syndrome-like phenotypes, pregnancy loss, and low birth weight. With widespread human dietary exposure and growing experimental evidence of endocrine-disrupting properties, a comprehensive review of the impact of ZEN, zeranol, and their metabolites on the female reproductive system is warranted. The objective of this systematic review was to summarize the in vitro, in vivo, and epidemiological literature and evaluate the potential impact of ZEN, zeranol, and their metabolites (commonly referred to as mycoestrogens) on female reproductive outcomes. We conducted a systematic review (PROSPERO registration CRD42020166469) of the literature (2000–2020) following the Preferred Reporting Items for Systematic Reviews and Meta-Analyses (PRISMA) guidelines. The data sources were primary literature published in English obtained from searching PubMed, Web of Science, and Scopus. The ToxR tool was applied to assess risk of bias. In vitro and in vivo studies (*n* = 104) were identified and, overall, evidence consistently supported adverse effects of mycoestrogens on physiological processes, organs, and tissues associated with female reproduction. In non-pregnant animals, mycoestrogens alter follicular profiles in the ovary, disrupt estrus cycling, and increase myometrium thickness. Furthermore, during pregnancy, mycoestrogen exposure contributes to placental hemorrhage, stillbirth, and impaired fetal growth. No epidemiological studies fitting the inclusion criteria were identified.

## 1. Introduction

Zearalenone (ZEN) is a secondary metabolite of *Fusarium* fungi and is one of the most common mycotoxin contaminants in global food supplies [1]. ZEN contaminates cereal grains (e.g., maize, wheat, barley, oats and sorghum), and is widely detected in processed foods (i.e., pasta, breakfast cereal and bread) [1,2,3]. Several studies have documented ZEN concentrations in foodstuffs above established European Union (EU) maximum contamination limits (100–200 µg/kg for unprocessed cereals, 75 µg/kg for processed cereals) as summarized in a recent review [2]. At the same time, adverse reproductive health outcomes following mycoestrogen exposure have been reported in the animal husbandry literature for decades [4,5]. In swine, mycoestrogen exposure led to vulvo-edema, enlarged uteruses, sclerotic and atrophic changes to the ovary [5] and reduced fetal weight [4]. There has been limited research on the potential of mycoestrogens to impact human health. However, given mounting evidence of widespread mycoestrogen contamination in the food supply as well changes in climate promoting their growth, there is a clear need for additional investigation [6,7,8].

**Figure 1:**
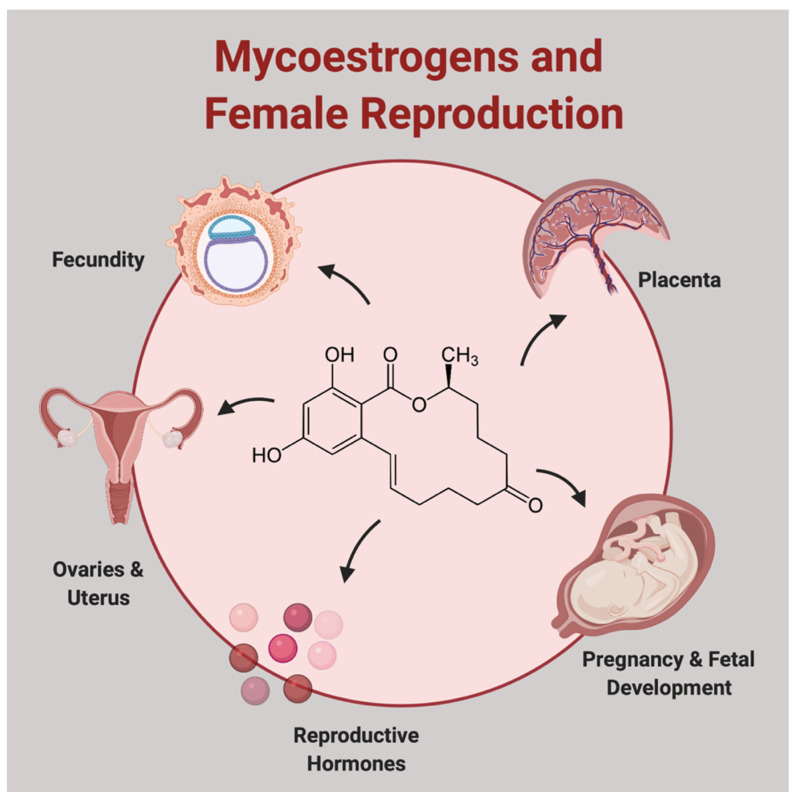
Impact of mycoestrogens on female reproduction. Created with BioRender.com.

**Figure 2:**
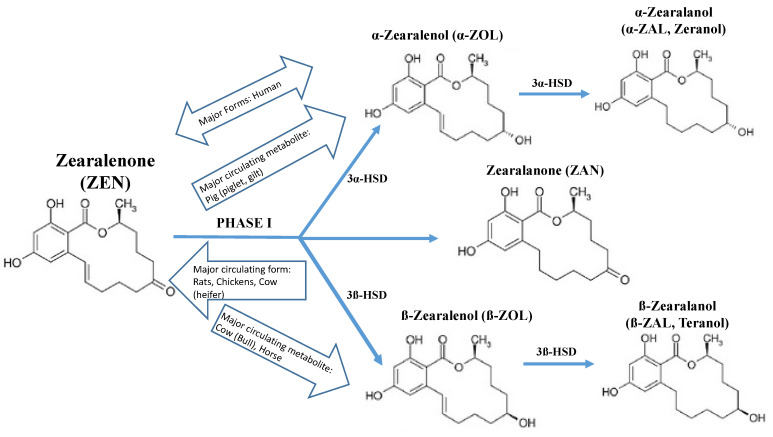
Phase 1 biotransformation of zearalenone (ZEN) by species. ZEN is metabolized by hydroxysteroid dehydrogenases (HSD) into biologically active metabolites, α-ZOL and β-zearalenol (β-ZOL) as well as zearalanone (ZAN). α-ZOL is further reduced to form zeranol (α-zearalanol).

Impacts on reproductive health endpoints likely occur through ZEN’s ability to alter estrogenic signaling (Figure 1). Its chemical structure closely resembles 17β-estradiol (E_2_), which enables direct binding to the nuclear estrogen receptors α (ERα) and β (ERβ) [9]. Notably, however, the structures of ZEN and zeranol lack the sterol backbone and as a result are not considered steroids. Nonetheless, in ligand-activated receptor binding experiments, the ZEN–ERα complex bound to the human estrogen response element (ERE) with nearly the same binding affinity as the E_2_–ERα complex (K_d_ of E_2_ = 32 nM; K_d_ of ZEN = 34 nM); similar results were observed for ZEN–ERβ (K_d_ of E_2_ = 84 nM; K_d_ of ZEN = 70 nM) [10]. Likewise, the ZEN metabolite, α-zearalenol (α-ZOL), is nearly as estrogenic as estradiol (EC_50_ = 0.022 nM α-ZOL; EC_50_ = 0.015 nM E_2_) and 70-fold more potent than ZEN, as demonstrated in human reporter gene assays [11]. Other ZEN metabolites can also bind ERα/β (Figure 2). Thus, ZEN’s ability to directly bind to estrogen receptors may explain its greater estrogenic potency compared to other known endocrine disruptors [E_2_> ZEN > genistein > bisphenol A (BPA) > dibutyl phthalate (DBP) > di(2-ethylhexyl) phthalate (DEHP)] [12,13,14]. The estrogenic potency of mycoestrogens has also been demonstrated in vivo. For example, in mice, rats, and pigs, ZEN and metabolites readily bind uterine ERα and ERβ receptors [15,16,17,18]. Pig studies further suggest that zeranol has a higher affinity to ERα than ZEN or the metabolites α- and β-ZOL [17]. The ability of ZEN and its metabolites to bind to estrogen receptors earned them their designation as ‘mycoestrogens’. Although some literature suggests that other mycotoxins (e.g., alternariol) may act as mycoestrogens, for the purpose of the current review, we use the term mycoestrogen to refer solely to ZEN, zeranol, and their metabolites [19,20].

Due to their ability to influence estrogenic responses, mycoestrogens may affect the development and function of the female reproductive system by disrupting the hypothalamic–pituitary–gonadal axis (HPG). The HPG axis regulates gonadal secretion of sex steroids through sequential positive and negative feedback loops centered on hormone levels. For example, gonadotropin-releasing hormone (GnRH), secreted by the hypothalamus, stimulates the secretion of follicle-stimulating hormone (FSH) and luteinizing hormone (LH) by the anterior pituitary. FSH and LH stimulate the ovaries to secrete estrogen. Estrogen initiates a negative feedback loop and blocks the secretion of GnRH by the hypothalamus. During folliculogenesis, the maturation and development of oocytes, granulosa cells proliferate and function to secrete sex steroids (i.e., E_2_, progesterone (P_4_)) which support oocyte growth and development as well as HPG axis function [21]. Studies across species observed that ZEN impacts ovarian follicle development and function, resulting in significantly lower circulating E_2_ levels [22,23,24]. Lower E_2_ levels directly impact growth but may also alter LH and FSH concentrations, though direction of change (i.e., enhance or suppress) depends upon model organism, timing of exposure, and dose [21,25,26,27,28]. These discrepancies in responses may result from significant interspecies variability in biotransformation of ZEN and zeranol. It may also explain why the synthetic version of α-ZAL, zeranol is marketed as a growth promotor in the U.S. and Canadian livestock industries whereas experimental animal models have typically demonstrated impaired growth following prenatal exposure [22,29].

The primary route of human exposure to ZEN is by ingestion and its biotransformation has been well characterized in mammalian models. In pigs, 80–85% of a single oral dose (10 mg/kg bw) is absorbed and has an 86 h half-life [30], though absorption and half-life may be lower in other species [31]. Studies in several animal models show that after ingestion, ZEN is distributed mainly to the liver (pigs, sheep, cows, rats, mice, and hens) but also reproductive organs (rat, mouse, and pig) and the placenta (rabbits, rats, and humans) [32,33,34,35,36,37]. As was recently reviewed by Rogowska et al. (2019), ZEN follows two major biotransformation pathways—hydroxylation and conjugation—resulting in a variety of metabolites including the biologically active α-ZOL and β-zearalenol (β-ZOL) (Figure 2) [33]. Briefly, in the liver, 3*α-* and 3β-hydroxysteroid dehydrogenases (HSD) convert ZEN to α-ZOL, β-ZOL, and zearalanone (ZAN) [38]. The predominant Phase 1 metabolite in pigs is α-ZOL, whereas β-ZOL dominates in horse and bull [39]. Further reduction produces α-ZAL (zeranol) and β-ZAL, which can be further reduced to ZAN. Phase 2 metabolism of ZEN or ZEN Phase 1 metabolites by UDP-glucuronosyl transferases (UGTs) and sulfotransferases (SULTs) produces glucuronide and sulfate conjugates, respectively, that are secreted into bile or urine and thereby cleared from the body [33]. An early study of human volunteers administered a single oral dose of zeranol found the half-life was 22 h [40]. Humans produce both Phase 1 metabolites with formation of α-ZOL exceeding that of β-ZOL [31].

Given the prevalence of mycoestrogens in the food supply and their potential to disrupt endocrine signaling, 16 countries have enacted regulations to limit human exposure [3]. In the EU, zeranol is banned and the tolerable daily intake (TDI) for ZEN is 0.25 μg/kg/bw, with concentration limits set at 100–200 μg/kg for unprocessed cereals and 75 μg/kg for processed cereals. Meanwhile, in the U.S., the acceptable daily intake (ADI) for zeranol is 1.25 μg/kg/day and the United States Food and Drug Administration (US FDA) monitors but does not set an advisory level for ZEN in the food supply [41,42,43,44]. To assess human exposure, mycoestrogens are typically measured in urine using liquid chromatography coupled to a mass spectrometer [45]. A recent systematic review of mycotoxin biomonitoring reported that ZEN was detected in every population studied across multiple continents including Africa (Cameroon, Nigeria, South Africa), Europe (Belgium, Italy, Germany, Sweden), Asia (Bangladesh), and the Americas (Haiti) [46]. Other studies have also reported detectable levels in the U.S. and Brazil [47,48,49]. Exposure levels ranged from picograms to nanograms depending on (1) type of biospecimen (plasma, serum, maternal breast milk, and urine), (2) limit of detection for each assay, and (3) geographical region [46]. Although most studies have observed human exposures to generally be below the TDI, a recent human health risk assessment of ZEN estimated that the maximum upper bound of ZEN exposure (95th percentile) exceeded the TDI by a factor of 1.7 for infants and 2.2 for toddlers [50]. Notably, only a small number of human studies have examined mycoestrogen exposure in relation to human health outcomes. Of these, the vast majority focused on peri-pubertal exposure and all reported changes in growth and/or pubertal timing associated with elevated ZEN concentrations [48,51,52,53,54].

Prior publications have reviewed potential endocrine-disrupting properties of ZEN, particularly in relation to sex steroid hormones and in vivo reproductive outcomes [9,33,55]. However, to our knowledge, there is no comprehensive review on the impact of mycoestrogens on female reproduction. The aim of this systematic review was to evaluate and summarize the current in vitro, in vivo, and epidemiological literature on mycoestrogen exposure (post-2000) in relation to female reproductive outcomes.

## 2. Results

**Figure 3:**
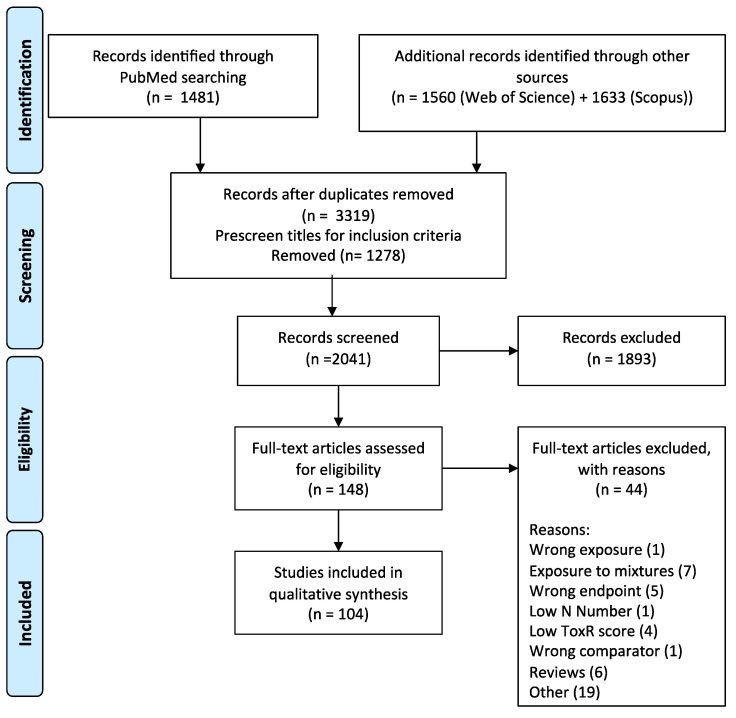
PRISMA flow diagram.

The literature search identified 4674 articles (1481 articles in PubMed, 1560 in Web of Science, and 1633 in Scopus), with 3319 remaining after duplicates were removed (see Figure 3 for complete PRISMA Flow Diagram). Title screening in Endnote eliminated 1278 articles that did not meet inclusion criteria outlined in the review protocol and PECO (person, exposure, comparator, outcome) statement (Table 1). 2041 articles were assessed by title and abstract in Rayyan QCRI, and 1893 were excluded because they did not meet inclusion criteria, full text articles could not be accessed (*n* = 6) or were published prior to 2000. In total, 148 papers were read for full text eligibility and reviewed for RoB using ToxR, of which 104 studies were included in the narrative review based on Klimisch scores of 1 or 2 (ToxR scores for all reviewed full texts are provided in Supplementary Table S2). Model systems reviewed include in vitro studies in cell lines derived from humans, pigs, cows, rats, mice, hamster, sheep and horse, while in vivo studies ranged from rats, mice, pigs, dogs, cows and included a variety of routes of administration (contaminated feed, implants, oral gavage, intraperitoneal injection and in utero exposure.)

The narrative synthesis that follows details the evidence of the impact of mycoestrogens on reproductive hormone regulation in the non-pregnant state, the ovaries and uterus, fecundity/conception, pregnancy and the placenta, and birth outcomes. Detailed data tables are provided in Supplementary Tables S3–S7.

### 2.1. ZEN and Reproductive Hormone Regulation in the Non-Pregnant State

Thirteen studies examined HPG axis activity following mycoestrogen exposure with adverse effects evident across species (Table 2 and Table S3). Through direct binding to ER and estrogenic mimicry, mycoestrogens may act as a negative regulators of GnRH, thereby blocking release of LH from the anterior pituitary [21] as observed in a number of studies [23,25,28,56]. In vitro treatment of bovine anterior pituitary cells with zeranol, moreover, suppressed production of LH through a G protein-coupled receptor 30 (GPR30)-based mechanism [57]. This effect was noted only at relatively low concentrations (0.001 nM–1 nM), but not at 10 or 100 nM. Although the strongest GnRH-mediated suppression occurred in response to ZEN, its metabolites also reduced LH secretion from anterior pituitary cells (ZEN > α-ZAL > zearalanone > α-ZOL > β-ZOL > β-ZAL) [58]. By contrast, in an in vitro porcine anterior pituitary cell model, ZEN and α-ZOL inhibited FSH (but not LH secretion); reduction of FSHβ transcription was also mediated by the GPR30 and the effect may have occurred through binding sites for LIM homeodomain transcription factor LHX3 in the FSHβ promoter (LHβ lacks LHX3-binding sites) [59].

The in vitro observation that ZEN can impact reproductive hormones along a non-monotonic dose–response relationship has been supported by in vivo studies in cycling animals (Table 2). In 8-week-old mice, long-term exposure (30, 60 or 90 days) to 2.5 mg/kg bw ZEN reduced LH, FSH, E_2_, and P_4_ in serum [28]. Similar results were observed in pigs [23]. By contrast, exposure of mice to higher doses of ZEN (10 mg/kg bw) showed increased LH and decreased E_2_ production suggestive of ovarian failure [27]. Moreover, a study in gilts found that a decrease in serum LH following ZEN exposure was accompanied by an increase in E_2_ [56]. It has been suggested that this may be a compensatory mechanism by the anterior pituitaries to limit an overall increased in E_2_.

Although many early studies focused on high-dose mycoestrogen exposure through the livestock industry [4,5], more recently, models recapitulating the low-level chronic exposure through diet suggested by human biomonitoring studies have been increasingly employed. For example, after 3 months of low-dose (0.1–1 mg/kg bw) chronic ZEN exposure administered at mating age, numerous endocrine changes were noted in female rats in a dose-dependent manner [26]. Testosterone (T) and LH increased significantly in ZEN-treated rats compared to controls, whereas E_2_ and FSH levels were significantly lower. P_4_, insulin, and glucose all increased as well with greatest changes observed at a dose of 1 mg/kg bw [26]. By contrast, subchronic exposure to ZEN in young female rats fed a diet containing between 0.5 and 3.6 mg/kg for 4 weeks did not significantly alter FSH despite reductions in weight gain compared to controls [62]. Additional work in pigs suggested that ZEN induced changes in the peripheral blood concentrations of E_2_ at 10 and 15 µg/kg bw, but not the lowest dose (5 µg/kg bw); P_4_ and T were also depressed after 14 and 42 days of treatment [64]. The spontaneous secretion of E_2_ was low (4 to 9 ng/mL) following 20 days of exposure to 20 to 40 µg/kg bw ZEN [63]. Across all models, exposure to 10 µg/kg bw ZEN in the pig was the lowest dose that produced a statistically significant change in hormone levels [64]. In summary, significant disruption to circulating hormones was observed across species in nearly all studies following ZEN exposure even at low, human-relevant concentrations.

### 2.2. ZEN and the Ovaries

*In vitro studies*. We reviewed 24 papers on the impact of ZEN and its metabolites on ovarian folliculogenesis using in vitro and ex vivo cultures across multiple species (Table 3 and Table S4). Ex vivo exposure of neonatal mouse primordial follicles to 10 and 30 µM ZEN resulted in a concentration-dependent decline in the number of oocytes. Disruption of oocyte availability was accompanied by downregulation of transcriptional factors essential for primordial follicle formation including LIM homeobox 8 (*Lhx8*), factor in the germline alpha (*Figlα*), spermatogenesis and oogenesis helix-loop-helix (*Sohlh2*), and newborn ovary homeobox (*Nobox*) [70]. Across species (murine, porcine, bovine), granulosa cells within primordial follicles consistently exhibited dose-dependent decreases in proliferation and more extensive apoptosis following ZEN treatment (10—200 µM) [71,72,73,74,75,76,77]. Increased apoptosis may reflect ZEN-mediated dysregulation of the endoplasmic reticulum–mitochondria axis, as evidenced by greater induction of cleaved caspase protein levels [72,73,74]. By contrast, at significantly higher micromolar concentrations (10 to 50 µM), ZEN exhibits antagonistic properties and limits estrogen receptor activity [78]. In addition, numerous studies demonstrated significantly lower maturation rates and increased apoptosis of porcine and bovine oocytes following ZEN administration, with some differences observed in relation to the various metabolites evaluated [79,80,81,82]. Earlier studies indicate that the impacts on oocyte maturation occurred in the absence of degeneration and DNA damage [83], consistent with the evidence that ZEN is a potent estrogen, but not overtly cytotoxic [74]. However, more recent research shows that ZEN exposure leads to an increase in DNA double-strand breaks (DSBs) in ovine and murine models [24,84]. At lower doses (0.001 to 3.1 µM), administration of ZEN or its metabolite α-ZOL stimulated proliferation of bovine and mare primary granulosa cells [85,86]. The conflicting results concerning granulosa cell proliferation may be attributable to the concentration-specific ability of ZEN to impact estrogenic responses. At nanomolar concentrations, ZEN exerts distinct agonistic activity on ERα and ERβ. Collectively, the results of in vitro studies suggest that ZEN impairs the early stages of folliculogenesis through negative impacts on primordial follicle development and rates of oocyte maturation.

*In vivo studies*. Endocrine-disrupting compounds often target ovarian development and function. In the 25 in vivo studies reviewed, the most common endpoints included changes in the number of primordial follicles, estrous cyclicity, ovarian weight, and corpora lutea integrity (Table 3 and Table S4). Mycoestrogens have deleterious effects on ovarian development and function across a range of doses and species, particularly following gestational exposure. Treatment of mice with 20 to 40 µg/kg/d ZEN from gestational day (GD) 12.5 to 15.5 resulted in altered meiosis in fetal oocytes at GD 15.5, with higher and lower percentage of germ cells in zygotene and diplotene stages, respectively [24]. The mitotic changes parallel the significantly lower number of primordial follicles observed in newborn mouse ovaries following ZEN exposure [24].

Evidence indicates that ovarian impacts following gestational exposure to mycoestrogens extend beyond folliculogenesis. In mice and rats, in utero exposure to ZEN (at concentrations ranging from 20 µg/kg/d–40 mg/kg/d) caused prolonged duration of estrus, lack of corpora lutea, and fewer functional follicles in F1 generation females [24]. Animals exposed to ZEN as neonates or post-weaning also spent more time in estrus compared to controls [25,90,92]. Impacts on ovarian cycling have been noted in livestock as well with heifers treated with zeranol implants exhibiting delayed cyclic reproductive tract development according to morphological scoring [105].

Mycoestrogen-mediated insults on ovarian development are also observed in prepubertal and pubescent exposure models. In mouse, rat, and porcine species, ZEN administration to prepubertal females (at 200 µg/kg/d–10 mg/kg/d) accelerated onset of puberty, decreased the number of primordial follicles, increased follicular atresia, and ovarian weight, and prolonged the duration of estrous (Table 3 and Table S4). ZEN administration (2.5–10 mg/kg/d) during the prepubertal period may also result in prolonged anovulatory ovaries in mice and rats as depicted by slower development of corpora lutea [90]. Additionally, prepubertal treatment with low-dose ZEN (20–40 µg/kg/day) dramatically reduced the number of primordial follicles. In adult mice, ZEN administration (0.1 to 2.5 mg/kg/d) decreased the number of mature follicles and enhanced follicular atresia [28,91]. The absence of corpora lutea noted in adult mice following a 30 day treatment with 2.5 mg/kg bw ZEN suggests significant disruption of oocyte maturation [28].

We observed evidence of inter-species differences in ovarian sensitivity to mycoestrogens. Porcine models appear to be most sensitive with ovarian folliculogenesis significantly impacted by ZEN at doses as low as 20 to 200 µg/kg/d [99,106]. By contrast, in rats, exposure to higher 0.5 to 1 mg/kg bw doses have inconsistent impacts on estrous cycling, with few changes in ovarian weight and morphology noted [26,87,92,93]. It has been postulated that compared to other species, swine produce higher levels of the more potent estrogenic ZEN metabolite, α-ZOL, resulting in greater sensitization [32,42]. Moreover, the relative binding affinity of α-ZOL to estrogen receptors differs across species and is stronger in pigs relative to rats (reviewed by Metzler, Pfeiffer [107]). Overall, expression levels of ERα and ERβ vary by species and tissue, in the mammalian ovary germinal epithelium mycoestrogens appear to be full agonists of ERα, and in ovarian granulosa and cumulus cells partial agonists of ERβ [108]. Human sensitivity to mycoestrogens as a reproductive toxicant has not yet been examined, nevertheless, the results of in vitro and in vivo studies implicate mycoestrogens as a reproductive toxicant causing significant dysregulation of ovarian folliculogenesis [99,106].

### 2.3. ZEN and the Uterus

Studies examining uterine outcomes after mycoestrogen exposure (*n* = 28) have focused on three topics: (1) receptor binding (*n* = 4); (2) gene expression, cell viability and apoptosis (*n* = 6); (3) morphological and structural changes (*n* = 17) (Table 4 and Table S5).

*Receptor-binding studies.* Molecular studies in pigs, rats, and mice examining mycoestrogen exposure and receptor binding showed binding to ERα and ERβ in the uterus [15,16,17,18]. Some evidence in rats suggests impacts of ZEN on estrogen receptor density in the uterus as well [16].

*Gene expression, cell viability, and apoptosis.* A smaller set of studies (*n* = 6) examined molecular and cellular changes in the uterus following mycoestrogen exposure. For example, in a small study *(n* = 2) of adult heifers, zeranol treatment (36 mg implants) decreased the expression of uterine muscle function and energy metabolism genes (e.g., *ACTA1*, *HK*) as well as *BCL-2*, a major inhibitor of cell death [122]. By contrast, in post-weaning gilts, Zhou, Yang [115] reported linear increases in uterine BCL-2 mRNA expression following ZEN exposure. An in vitro study in mouse endometrial stromal cells further suggested BCL-2 dysregulation in a concentration-dependent manner (0–100 µM), reporting an increase in the BAX/BCL-2 ratio after ZEN treatment [120]. While BCL-2 guards against apoptosis, BAX is a promoter of apoptosis and a higher BAX/BCL-2 ratio favors a greater rate of apoptosis. Xie, Hu [118] also reported decreased cell viability in mouse endometrial stromal cells after ZEN exposure (>50 µM). Other cytotoxicity markers were enhanced including cell shrinkage, disruption of the cell cycle, and differential expression of genes related to cell cycle and apoptosis such as *Bec-2 family*, *Cdc* genes and *Hoaxa-10*. The ZEN metabolites, α- and β-ZOL, have also been linked to decreased cell viability and impaired ability to proliferate in porcine granulosa cells [101,119]. In pig endometrial stromal cells, β-ZOL altered transcription of cell cycle-dependent kinases and reduced phosphorylation of MAPK and AKT (PKB) kinases [119]. Additionally, recent studies in porcine endometrial cells indicate that ZEN can activate the WNT/β-catenin signaling pathway to promote proliferation [123]. Overall, the evidence from murine models points toward strong apoptotic effects of mycoestrogens in the uterus and decreased cell viability in endometrial stromal and granulosa porcine-derived cells [101,118,120].

*Morphological and structural changes.* Of 17 studies examining uterine weight, all but one reported significantly increased uterine weight in ZEN-treated animals compared to controls. The majority of these studies were conducted in pre-pubertal or peri-pubertal animals (mostly mice and rats) while the one study reporting a decrease in uterine weight was a study conducted in mature rats [61]. Thickening of uterine layers (endometrium, myometrium, and perimetrium), a characteristic of pubertal development, was observed following ZEN exposure (doses > 1 mg/kg) in three studies using pre-pubertal or pubertal experimental animals (rat and pig) [56,92,115]. Moreover, simple glandular hyperplasia of the endometrium was observed with doses as low as 75 µg/kg bw in prepubertal bitches [114]. By contrast, in mice and rats with higher ZEN exposure (>10 mg/kg) during gestation or shortly after birth (0–7 days) resulted in thinning of uterine layers [68,110], suggesting that as with other endpoints, ZEN’s impacts on uterine development may vary based on the timing of exposure and dose. Prenatal and early post-natal ZEN exposures were also associated with delayed uterine development as marked by fewer endometrial glands and infiltrating eosinophils [124].

### 2.4. ZEN and the Placenta

*Disposition in the placenta.* The placenta is a key determinant of fetal health by secreting hormones necessary for maintaining pregnancy, exchange of nutrients, gases, and wastes, attachment to the uterus, and transfer of xenobiotics to the fetal circulation. As a result, the placenta regulates the disposition of chemicals and their metabolites within the fetoplacental unit. An early study demonstrated that zeranol residues could be detected in the placentas of Dutch-Belted rabbits [37] but a comprehensive characterization of zeranol disposition has not been performed. Rather, the evidence of mycoestrogen transfer across the placenta has focused on ZEN (Table S6). Following a single injection to Sprague Dawley rats, ZEN and the α-ZOL metabolite could be detected in placentas and fetuses, although at concentrations significantly below levels observed in maternal livers [34]. Interestingly, higher concentrations of ZEN were observed in rat placentas at GD 12 compared to 18. These data suggested that while ZEN can partition into and cross the placenta, there may be cellular transporters that limit its transfer to the fetus [125,126]. Recent studies have demonstrated ex vivo ZEN transfer in dually perfused human placentas further supporting the ability of mycoestrogens to enter and cross the placenta [36].

*Placental signaling and health.* In addition to, identifying mechanisms by which the placenta regulates the transfer of mycoestrogens to the fetus, it is important to examine the direct impact of ZEN and zeranol on critical placental functions including cell fusion, hormone secretion, and placental barrier maintenance. Within the human placenta, mononucleated cytotrophoblasts fuse to form multinucleated giant cells that are called syncytiotrophoblasts. These syncytiotrophoblasts constitute the outermost later of placental villi and are in direct contact with maternal blood. Interestingly, ZEN (10 μM) increases the process of cell fusion, resulting in greater numbers of multinucleated BeWo cells, a human placental cell line derived from a choriocarcinoma [127]. Stimulation of fusion by ZEN coincides with the increased expression of *Syncytin 1/2*, key genes involved in syncytialization, influenced by the cAMP, MAPK, and Wnt signaling pathways [128].

To varying degrees, α- and β-ZOL also increased expression of Syncytin 2 and led to accumulation of cyclic AMP, a key signaling molecule responsible for cell fusion. As a result of stimulated cell fusion in response to ZEN exposure, a corresponding increase in the secretion of human chorionic gonadotropic (hCG) into media has been reported [127,128]. Using a pharmacological antagonist, it was further demonstrated that upregulation of hCG secretion occurred through an ER-dependent mechanism [127,128]. Computational modeling simulations have also suggested that mycoestrogens may bind the ligand-binding domain of the P4 receptor as well, which may be important for some of the cellular responses to ZEN [128].

The placenta is also a key regulator of the timing of parturition including the production of corticotropin-releasing hormone (CRH) and prostaglandins important for the rupture of fetal membranes. Interestingly, zeranol (up to 100 nM) exposure leads to the upregulation of CRH mRNA and protein in JEG-3 choriocarcinoma cells [129]. Similarly, zeranol enhances the expression of transient receptor potential channels (TRP) that regulate ion concentrations within cells, most notably calcium concentrations [130]. This event appears to be important for the subsequent upregulation of cyclooxygenase-2 (COX-2), an enzyme responsible for production of prostaglandins and thromboxanes from arachidonic acid. Taken together, enhanced production of CRH and COX-2 by zeranol may be important for risk of preterm labor.

Preliminary studies have also explored the impact of mycoestrogens on placental development in rodents and largely suggest that significant adverse responses are limited to high doses (Table 5). Administration of zeranol (1–100 mg/kg/d oral gavage) to pregnant ICR mice from gestation day 13.5 to 16.5 increased mRNA expression of receptors for P_4_ and CRH (notably, at the 100 mg/kg/d dose level) [129]. These data correspond to in vitro studies with human cells, suggesting that mycoestrogens prime the placenta for heightened hormonal signaling. In a separate study, administration of zeranol (0.8–40 ppm in diet) to mice from gestation day 5.5 to 13.5 resulted in accumulation of neutral lipids including triglycerides within the placenta labyrinth layer, which may have sequestered lipids from transfer to the fetus [29]. In rats, reduction in relative mRNA abundance of ERα, GnRH receptor, and ATP-binding cassette transporters b1 and c1 (Abcb1 and Abcc1) accompanied 10 and 20 mg/kg exposure, but upregulated ABCc5 was observed at 20 mg/kg [67]. At high dietary concentrations of 40 ppm, exposure to ZEN reduced maternal weight gain and weights of the placentas (as well as fetuses). Furthermore, ZEN (40 ppm) reduced the area of placental layers within mice and caused hemorrhage [29]. While these observations at high dietary doses of mycoestrogens are concerning, few overt changes to the placenta have been reported at lower, environmentally-relevant concentrations.

### 2.5. ZEN and Fertilization, Pregnancy, and Fetal Development

*In vivo studies*. Given the widespread and long-standing use of zeranol in animal husbandry, its adverse impacts on fertility have long been recognized [142,143]. More recent research with model organisms confirms and extends these findings suggesting differential impacts depending on dose and timing of exposure (Table 5). Across a range of doses in mice and rats, ZEN administration during early gestation results in reduced maternal weight gain in mid-late pregnancy in most studies [22,67,133]. Not surprisingly, these maternal impacts are accompanied by extensive effects on pregnancy and fetal development including reduced number of pregnancies, more fetal deaths, more litter resorptions, and a reduced number of implanted, viable fetuses per litter, with more extensive toxicity demonstrated at higher doses [22,67,133]. For example, in a mouse model, ZEN administration on GD 1–5 (at doses ranging from 2 to 8 mg/kg bw) reduced rates of implantation and lowered the proportion of live fetuses. At the highest dose tested (8 mg/kg), the decidual response was entirely blocked [131]. Similar reductions in implantation were observed following pre-conception exposure, whereby only 1 of 11 ZEN-treated mice (40 ppm diet) had implantation sites (compared to 9/12 in the control group) [117].

It has been hypothesized that reductions in viability following ZEN administration may be the result of disrupted hormone production inhibiting embryo migration through the oviducts, thereby leading to delayed implantation [131]. Indeed, evidence from pregnant animals suggests disruption of several hormone pathways after ZEN exposure. In late pregnancy, prolactin (PRO) production increased in a dose-dependent manner in ZEN-treated rats compared to controls (2, 4, and 8 mg/kg bw), while P_4_ and E_2_ production decreased [66]. At higher doses (50, 100, 150 mg/kg diet), rats treated with ZEN early during pregnancy had decreased P_4_ and E_2_ concentrations, while levels of LH, FSH, and PRO were significantly increased in only the 100 and 150 mg/kg groups [22]. By contrast, in mice, late gestation exposure to zeranol at a range of doses (1, 10, 100 mg/kg) significantly elevated P_4_ at the highest dose, with no change in E_2_ concentrations [129]. In addition, reductions in testosterone levels were reported at all dose levels examined.

Given the extensive changes in maternal physiology in pregnancy after mycoestrogen exposure, it is not surprising that the growth of the resulting fetuses is impacted as well. Reduced live fetal weights and shorter crown-rump lengths has been noted, particularly at higher doses of ZEN [22,67,131]. Limited evidence suggests that these reductions in fetal size may occur in the absence of changes in placental size [131]. Fetal skeletal impacts including delayed ossification and anomalies have also been noted [67,133] and one study reported reduced fetal brain weights following higher-dose exposure (≥9 mg/kg bw/d) [22].

Our review included three studies of gestational exposure to zeranol [105,129,134]. In the first, gilts who were implanted with zeranol at puberty went on to have pregnancies with fewer and smaller fetuses, and a lower rate of fetal survival [134]. In heifers implanted with zeranol in adulthood, moreover, pregnancy rates (both overall and following artificial insemination) were lower in zeranol-treated animals as compared to controls. However, the differences were non-significant and the outcomes of the pregnancies were not reported [105]. Finally, pregnant ICR mice administered zeranol during late pregnancy (GD 13.5–16.5) had increased rates of fetal resorption and preterm birth and the average number of live newborn pups per dam was significantly reduced in the 10 and 100 mg/kg treatment groups [129].

*In vitro studies*. In vitro studies (Table 5) have examined the mechanisms by which mycoestrogen exposure may impair fecundity and fertility. In ovary cumulus-oocyte complexes (COCs) from female pigs, exposure to α- and β-ZOL, or ZEN prior to fertilization resulted in roughly a 50% reduction in blastocyst formation compared to controls [135]. Exposed oocytes were more likely to develop into aneuploid blastomeres and results were consistent across doses (0.312–31.2 µmol/L) and also observed with exposure to metabolites. Other studies have reported dose–response relationships. Nazar, Lee [137] observed reduced embryo cleavage and blastocyst formation in bovine oocytes following exposures to α- and β- ZOL, with stronger effects observed at the higher 30 µmol dose [137]. ZEN exposure in porcine blastocysts lead to apoptosis, DNA damage, and autophagy [138,139]. Similarly, in porcine embryos, a dose–response relationship was observed between α-ZOL administration and cleavage rates starting at 10 µM, with decreased blastocyst development noted at 30 µM [136]. Other studies in gilt zygotes have noted similar effects at slightly lower α-ZOL concentrations (15 µM) [81]. By contrast, in one study of porcine oocytes, high-dose ZEN exposure (1000 µg/L) during in vitro fertilization had an unexpected positive effect on fertilization rates [80]. Overall, these results are suggestive of reduced embryo viability following exposure to ZEN and its metabolites, with stronger effects observed at higher doses. Additionally, studies indicate an increase in embryotoxicity, by administering ZEN to human embryonic stem cells (ESC) coincident with an increase in reactive oxygen species and apoptosis [140]. Overall, strong dose-dependent embryotoxicity was observed including reduced ESC survival and growth capacity, as well as increased generation of reactive oxygen species and apoptosis.

## 3. Discussion

This comprehensive systematic review provides consistent evidence that mycoestrogens adversely affect the ovary and uterus and contribute to changes in circulating hormones, the placenta, and adverse pregnancy outcomes. Mycoestrogens impair ovarian folliculogenesis and predispose the uterus to pathological and morphological changes following exposure. The differential responses of various reproductive organs to mycoestrogens may reflect the extent of ER isoform enrichment and affinity for binding ZEN and its metabolites. ERα is primarily expressed in the uterus, whereas ER*β* is primarily expressed in the ovary. Both isoforms are co-expressed in a number of tissues including the adrenal glands and some areas of the brain [144]. Tissue-specific differences in ZEN toxicity may be attributable to the endogenous expression of ER isoforms and the relative binding affinity of ZEN as well as their potential to act as agonists (partial or full) or antagonists [10]. Our findings suggest that the timing of exposure also dictates the sensitivity of reproductive organs to ZEN-mediated toxicity, with in utero and pre-pubertal exposures being most detrimental, similar to human sensitivity to exogenous estrogens [145]. In both the uterus and ovary, changes in morphology were also observed after pre-pubertal exposures to ZEN at environmentally-relevant doses [25,112]. Moreover, studies across species observed changes in ovary size, the density of oogonia, and the number of primordial follicles [81,90,96].

*Strengths and Limitations.* To our knowledge, this is the first systematic review to examine the effect of mycoestrogens on female reproduction. The core strengths of this project are the protocol aligned with the PRISMA guidelines, and comprehensive, structured search criteria to capture relevant publications. We used a widely accepted risk of bias tool to evaluate studies (ToxR tool). The review considers a wide variety of endpoints relevant to female reproduction, across several mammalian species. The drawback of this approach is that the variation between studies and species disqualifies for meta-analysis. Health endpoints influencing female reproduction, such as obesity and metabolism, were outside the scope of this review but should be considered in the future.

Due to the ability of mycoestrogens to alter estrogenic responses, this review focuses primarily on adverse outcomes in females. Human epidemiological studies have reported that ZEN exposure maybe linked to precocious puberty in girls [48,51]. Given the paucity of mycoestrogen-related epidemiological research and reproductive and birth outcomes in humans, in vivo and in vitro models were used to assess the biological plausibility of negative health impacts due to mycoestrogen exposure. Most papers (*n* = 89) received a top Klimisch score indicating ‘reliable without restrictions’, and the rest of the papers included (*n* = 15) scored Klimisch 2, indicating ‘reliable with restrictions. Papers receiving Klimisch 3 (*n* = 4) were excluded as a result of losing points for study results documentation when the study results for endpoints investigated were not transparent and/or complete. Additionally, studies where the design chosen did not allow for obtaining substance-specific data were excluded and deemed unreliable.

Animal models allow scientists to interrogate specific questions regarding timing of exposure, dose and mechanism of toxicity; however, they do not recapitulate human responses due in part to intraspecies variation in metabolism, exposure, and molecular signaling pathways. Ideally, to be conservative, the most sensitive species should be employed in research investigating the potential of a compound to act as an endocrine disruptor to limit false-negative results and identify most hazards [146]. Some of the doses used in the experiments considered in this review were higher than typical human exposure. Additionally, animal studies to date do not reflect the effect of multiple endocrine-disrupting chemicals (EDCs) exposure found in human populations and it is plausible that mycoestrogens may interact with other common EDCs (e.g., phthalates, BPA, genistein) as well as naturally co-occuring mycotoxins (e.g., deoxynivalenol, aflatoxin B1) to impact reproductive health in synergistic or antagonistic ways.

*Research Gaps and Future Directions.* We have identified key areas for future research. First, while some studies have utilized doses of mycoestrogens that recapitulate human exposure, additional animal studies are needed to assess the impact of exposure to chronic low doses as well as mixtures of mycoestrogens and other endocrine disruptors. Second, additional work on mycoestrogen exposure across the lifespan is needed to assess specific windows of exposure, cumulative damage of mycoestrogens, “multi-hit” models, and consequences of maternal mycoestrogen exposure for offspring beyond birth outcomes. Third, validation of most appropriate animal model for various endpoints would maximize the translational potential of the research. Indeed, given the preponderance of in vitro and in vivo literature on mycoestrogens, the primary gap in the current literature is the lack of epidemiological literature. Priority areas of epidemiological studies are informed by the animal literature and include (1) prenatal exposure to mycoestrogens and its impact on birth outcomes, particularly fetal and infant growth; (2) the impact of mycoestrogens at hormonal and developmental sensitive time points: in utero, early childhood, and puberty; (3) the impact on fecundity and fertility; and (4) mycoestrogen exposure in mixtures with other EDCs.

## 4. Conclusions

The current literature offers compelling evidence that mycoestrogen exposure affects female reproductive organs and hormone activity. Often, differential responses are observed based upon the dose and timing of exposure. Notably, adverse fetal outcomes were mostly observed at the higher doses tested in animal studies. Given the widespread exposure to mycoestrogens in the food supply and its consequences for female reproduction in experimental studies, it is imperative to conduct epidemiological studies of the impact of mycoestrogens on human health. Key attention should be placed on sensitive time points, such as development and pregnancy, and in populations vulnerable to high exposure, such as children and communities with high consumption of contaminated food products.

## 5. Materials and Methods

A systematic literature review was performed to identify studies of exposure to mycoestrogens in relation to female reproductive outcomes. We developed a PECO statement describing key elements (population, exposure, comparators, outcomes) (Table 1) The review was conducted according to the PRISMA guidelines [147]. The PRISMA checklist is provided in Supplementary Table S8. The protocol was registered with PROSPERO (20 January 2020), the International Prospective Register of Systematic Reviews (CRD42020166469) and it can be accessed at https://www.crd.york.ac.uk/prospero/display_record.php?RecordID=166469 accessed on 23 May 2021. 

*Eligibility criteria, information sources, search*. The literature search was limited to primary, English language literature published between 1 January 2000 and 31 December 2020. The literature search was repeated prior to submission and again, prior to final publication to capture any newer publications. The databases PubMed, Web of Science, and Scopus were searched using a strategy based on terms related to pregnancy, reproductive hormones, fertility, ovary, uterus, and mycoestrogens. The exposure terms included ZEN, ZEN metabolites and analogs: α-ZOL, β-ZOL, zeranol, and zearalalone (ZAN). Outcome search terms included premature birth, gestation, embryo, fetal, maternal, birth weight, prenatal, antenatal, reproduction, perinatal, utero, junctional zone, giant trophoblasts, spongiotrophoblasts, synctiotrophoblasts, spongiotrophoblsts, *Syncytin 1*, *Syncytin 2*, *Gcm1*, *Gcma*, Labyrinth zone endocrine disruptor, hormone, estrogen, progesterone, estradiol, testosterone, hyperestrogenism, corticotropin-releasing hormone (CRH), luteinizing hormone (LH), follicle-stimulating hormone (FSH), pituitary gland, androgen, sex steroid, steroidogenesis, ovaries, follicles, uterus, fertility, and fecundity. Medical Subject Heading (MeSH) terms were incorporated when available and hand-searched articles from reference lists of included studies were also considered. Publication type was restricted to journal articles and the search was restricted to titles and abstracts.

Inclusion criteria for in vivo studies with a sample size greater than 4 were female mammalian animal models with exposure to mycoestrogens at any life stage. Exposure to mycoestrogens included all range of doses, duration and routes of exposure including oral gavage, intraperitoneal injection, contaminated feed, or in utero exposure. Comparators for in vivo studies were exclusively vehicle-treated animals. Outcomes included were: changes in plasma or serum levels of hormones related to reproduction, pathological changes in ovaries or uterus, changes in weight of ovary or uterus, fertilization rates, pregnancy rate, fetal body weight and length, and placental hemorrhage. In vivo and ex vivo studies included cell lines derived from female reproductive organs and organs of the hypothalamus–pituitary axis. Exposure to mycoestrogens at all range of doses was considered and cells receiving vehicle only were the comparator. The included outcomes for in vitro experiments were: oocyte maturation rates, granulosa cell proliferation, cell viability, ROS production, apoptosis, cell viability, ZEN metabolite conversion, DNA damage, autophagy, blastocyst formation rate, embryo cleavage, blastocyst formation, embryotoxicity. The complete search strategy in can be found in Supplementary Table S1.

*Study selection and risk of bias*. Initial search results were pooled in a library within EndNote X9 software (Thomson Reuters, New York, NY, USA); duplicates were removed using the software and manual review. Titles were screened and articles that were outside the scope of the review were excluded (e.g., exposure to combinations of chemicals, non-mammalian species, analytical method development and validation, exposure assessments). Titles and abstracts of remaining articles were uploaded to the web application Rayyan QCRI (Qatar Computing Research Institute, Hamad Bin Khalifa University, Doha, Qatar), where titles and abstracts were screened by two independent reviewers; discrepancies were resolved by discussion or input from a third reviewer. The full text of remaining articles was downloaded and checked carefully for final inclusion criteria prior to evaluation using ToxRTool, a risk of bias (RoB) tool designed specifically for use in toxicological studies. Our search did not identify any epidemiological studies meeting inclusion criteria; therefore, a RoB tool intended for use in that literature was not needed.

The software-based ToxRTool, developed by the European Commission and used widely since 2009, was used to assess the reliability of in vivo and in vitro data [148]. The ToxRTool includes 21 evaluation criteria for in vivo studies and 18 criteria for in vitro studies; criteria fall into five categories including test substance identification, test system characterization, study design description, study result documentation, and plausibility of study design and results. For a given study, each criterion is assigned either a ‘1’ for ‘criterion met’ or a ‘0′ for ‘criterion not met’. Following ToxRTool specifications, in vivo studies that scored between 18 and 21 were considered Klimisch Category 1, 13–17, were Klimisch Category 2, and <13 were Klimisch Category 3 [148]. For in vitro studies, scores between 15 and 18 were Klimisch Category 1, between 11 and 14 were Klimisch Category 2, and <11 were Klimisch Category 3. For both in vitro and in vivo studies, Category 1 (reliable without restrictions) and Category 2 (reliable with restrictions) were included in the narrative synthesis section, and Category 3 (not reliable) were excluded. For RoB analysis, one reviewer (CK) reviewed 90% of articles, two reviewers (LA and LG) reviewed 10% of articles each, and 5% of articles were reviewed by two reviewers (LA and CK or LA and LG) to ensure inter-rater reliability.

*Data collection and synthesis of results*. Data items extracted from each article included first author, year of study, species, sex, strain, cell type, compounds studied, dose or route of exposure, timing of exposure, N number, systems, organs, and endpoints studied, positive control (if any), and main findings. Dose is reported as mg/kg bw/d for dosing and contaminated food exposures. Contaminated feed exposures are also reported by indicating the concentration of contaminant in feed and specifying ‘diet’; for example, ’40 ppm diet’ indicates 40 ppm diet per os. The great variation in species, dose, and route of administration across studies precluded a meta-analysis. Data tables were reviewed by all authors and summarized to provide a narrative synthesis of the literature.

## Figures and Tables

**Table 1:** PECO statement.

Study Type	Population	Exposure	Comparators	Outcomes
**In vivo studies**	Any female mammalian animal model, age, or life stage at exposure or outcome assessment.	Exposure to mycoestrogens at all ranges of doses, duration, and routes of exposure. Excluded: exposure to mixtures of chemicals and unmeasured doses of mycoestrogens.	Experimental animals receiving vehicle-only treatment.	Reproductive hormone levels, ovary and uterine weight, morphological and pathological changes in ovary or uterus, oocyte maturation rate, duration of estrus cycle, placental changes, implantation rate, pregnancy rate, gestational weight gain, resorbed/dead fetuses, live birth rate, fetal growth.
**In vitro and ex vivo studies**	Cells lines derived from ovaries, uterus, and anterior pituitary; and zygotes, blastocysts, embryos.	Exposure to mycoestrogens including all ranges of doses and durations. Excluded: exposure to mixtures of chemicals including mycoestrogens.	Cells receiving vehicle-only treatment.	Cell viability, reactive oxygen species, apoptosis, cell proliferation, fertilization rate, blastocyst formation and development, embryotoxicity, corticotropic-releasing hormone levels.

**Table 2:** Changes in circulating (plasma or serum) hormones following mycoestrogen exposure in the non-pregnant state and during pregnancy.

Outcome	LH *	FSH *	PRO *	P_4_ *	E_2_ *	T *	Experimental Model (Strain)	Dose (Compound, Route)	Age (Duration)	[Ref]
**Circulating Hormones During the Non-Pregnant State**	↓	↓			↓		Mouse (CD-1)	20 to 40 μg/kg (ZEN, IG)	4 wks (14 days)	[60]
	↓				↑		Mouse (BALB/C)	0.2 to 2 mg/kg (ZEN, SQ)	PND 1–5	[25]
	↑				↓		Mouse (BALB/C)	10 mg/kg (ZEN, IG)	3 wks (14 days)	[27]
	↓	↓		↓	↓		Mouse (Parkes)	2.5 mg/kg (ZEN, IP)	8 wks (up to 90 days)	[28]
	↑	↓		↑	↓	↑	Rat (Wistar Albino)	0.1 and 1 mg/kg (ZEN, IG)	9 wks (3 mos)	[26]
					NC		Rat (Sprague-Dawley, ovariectomized)	1 mg/kg (α-ZOL, IM)	4 wks	[61]
		NC					Rat (Sprague-Dawley)	0.5 to 3.6 mg/kg (ZEN, diet)	9 wks (28 days)	[62]
	NC	↓					Pig (NR, ovariectomized)	7.5 mg/kg (ZEN, IP)	NR, 24 h	[59]
					↑	↑↓	Pig (NR)	20 and 40 μg/kg (ZEN, capsule PO)	Pre-pubertal (48 days)	[63]
				↑↓	↑↓	↑↓	Pigs (NR)	5 to 15 μg/kg (ZEN, capsule PO)	Pre-pubertal (42 days)	[64]
	↓				↓		Pig (Landrace × Yorkshire)	200 to 1600 μg/kg (ZEN, diet)	Pre-pubertal (14 days)	[65]
	↓	↓	↑	↓	↓	↓	Pig (Landrace × Yorkshire × Duroc)	1.1 to 3.2 mg/kg (ZEN, diet)	2 weeks (18 days)	[23]
	↓	NC		NC	↑		Pigs (Duroc × Landrace × Large White)	1 mg/kg (ZEN, diet)	4 weeks (35 days)	[56]
**Circulating Hormones During Pregnancy**				↑↓	NC	↓	Mouse (ICR)	1 to 100 mg/kg (ZER, IG)	8 wks (GD 13.5–16.5)	[66]
	↓↑	↓↑	↓↑	↓↑	↓		Rat (Sprague-Dawley)	1 to 8 mg/kg (ZEN, IG)	NR (GD 6–19)	[67]
	↑	↑	↑	↓	↓		Rat (Sprague-Dawley)	0.3 to 146 mg/kg (ZEN, diet)	NR (GD 0–7)	[22]
		↑			↓		Rat (Sprague-Dawley)	5 to 20 mg/kg (ZEN, diet)	NR (GD 1–21)	[68]
	↓	↓					Rat (Sprague-Dawley)	5 to 20 mg/kg (ZEN, IG)	60 days (GD 14–21)	[69]

*: (statistically significant (*p* < 0.05) increase: ↑; or decrease: ↓; ↑↓: results differed by dose, timing, exposure or tissue collection; NC: no change). Abbreviations: α-ZOL: alpha-zearalenol; E2: estradiol; FSH; follicle-stimulating hormone; GD: gestation day; IG: intragastric; IM: intramuscular; IP: intraperitoneal; LH: luteinizing hormone; NR: not reported; PND: post-natal day; PO: per os; P4: progesterone; PRO: prolactin; SQ: subcutaneous; T: testosterone; ZEN: zearalenone, ZER: zeranol.

**Table 3:** Primary outcomes in the ovary following mycoestrogen exposure.

In Vivo Studies					
**Outcome**	Impact * (↑, ↓, ↑↓, NC)	Experimental Model (Strain)	Dose (Compound, Route)	Age (Duration)	[Ref]
**Ovary Weight**	↑	Rat (Sprague-Dawley)	0.3 to 146 mg/kg (ZEN, diet )	NR, GD 0–7	[22]
	NC	Rat (Sprague-Dawley)	0.2 and 10 mg/kg (ZEN, SC)	PND 15–19	[87]
	↑	Rat (Sprague-Dawley)	6 mg/kg (ZEN, diet)	3 wks (28 days)	[88]
	NC	Rat (Sprague-Dawley)	0.5 to 3.6 mg/kg (ZEN, diet)	3 wks (28 days)	[62]
	↑	Pig (Danbred)	0.75 mg/kg (ZEN, diet)	4 wks (21 days)	[89]
	↑	Pig (Duroc × Landrace × Yorkshire)	1.1 to 3.2 mg/kg (ZEN, diet)	2 wks (18 days)	[23]
	↑	Pig (Large White × Landrace × Pietrain)	6 mg/kg (ZEN, diet)	2 mos (26 days)	[88]
**Pathological or Morphological Changes in Ovary**	↑	Mouse (CD-1)	20 to 40 μg/kg (ZEN, SQ)	GD 12.5–18.5	[24]
	↑	Mouse (CD-1)	10 mg/kg (ZEN and ZER, SQ)	2 weeks (up to 24 wks)	[90]
	↑	Mouse (BALB/C)	0.2 to 2 mg/kg (ZEN, SQ)	PND 1–5	[25]
	↑	Mouse (BALB/C)	10 mg/kg (ZEN, IG)	3 wks (14 days)	[27]
	↑↓	Mouse (NR)	0.1 mg/day (ZEN, IG)	8 wks (10 days)	[91]
	↑	Mouse (Parkes)	2.5 mg/kg (ZEN, IP)	8 wks (up to 90 days)	[28]
	↑	Rat (Sprague-Dawley)	0.3 to 146 mg/kg (ZEN, diet ) (ZEN, diet)	GD 0–7	[22]
	↑↓	Rat (Sprague-Dawley)	5 to 20 mg/kg (ZEN, diet)	GD 0–20	[68]
	↑	Rat (Sprague-Dawley)	0.2, 1, 5 mg/kg (ZEN, IG)	PND 15–19	[92]
	↑	Rat (Sprague-Dawley)	0.1 to 10 mg/kg (ZER, SQ)	PND 15–19	[93]
	NC	Rat (Wistar Albino)	0.1 to 1 mg/kg (ZEN, IG)	9 wks (3 mos)	[26]
	↑	Rat (Wistar)	10 mg/kg (ZEN, IG)	PND 18 (10 days)	[94]
	↑↓	Pig (York × Finnish × Landrace)	200 to 1000 μg/kg (ZEN, diet)	GD 0–112	[95]
	↑	Pig (Duroc × Landrace × Yorkshire)	1.1 to 3.2 mg/kg (ZEN, diet)	2 wks (18 days)	[23]
	↑	Pig (Duroc × Landrace × Yorkshire)	1.04 mg/kg (ZEN, diet)	4 wks (35 days)	[96]
	↑	Pig (Duroc × Landrace × Large White)	0.5 to 1.5 mg/kg (ZEN, diet)	4 wks (35 days)	[97]
	↑	Pig (NR)	20 to 40 μg/kg (ZEN, capsule PO)	2 mos (48 days)	[98]
	↑	Pig (Large White × Landrace)	200 μg/kg (ZEN, capsule PO)	3 mos (8 days)	[99]
	↑	Pig (Large White × Landrace)	200 to 400 μg/kg (ZEN, capsule PO)	4 mos	[100]
**In Vitro Studies**					
**Outcome**	Impact * **(↑, ↓, ↑↓, NC)**	**Cell Type, Model (Strain)**	**Concentration (Compound)**	**Exposure Time**	**[Ref]**
**Cell Viability**	↓	Granular KK-1 Cells, Mouse (NR)	20 μM (ZEN)	24 h	[73]
	↓	Granulosa Cells, Mouse (Kunming)	15 to 150 μM (ZEN)	24 h	[72]
	↓	Granulosa Cells, Mouse (Kunming)	30 μM (ZEN)	24 h	[77]
	NC, NC	Granulosa Cells, Pig (NR)	5 to 30 μM (α-ZOL, β-ZOL)	48 h	[101]
**Reactive Oxygen Species**	↑	Granular KK-1 cells, (Mouse)	20 μM (ZEN)	24 h	[73]
	↑	Oocytes, Pig (NR)	5 to 30 μM (ZEN)	44 h	[82]
	↑	Ovaries, Sheep (NR)	1 μmol/L (ZEN)	3 days	[84]
**Oocyte Maturation Rate**	↓	Granulosa Cell, Mouse (ICR)	10 to 50 μM (ZEN)	24 h	[102]
	↓, ↓	Oocytes, Pig (Landrace)	3.5 to 90 μM (α-ZOL, β-ZOL)	48 h	[81]
	↓	Oocytes, Pig (NR)	1 to 1000 μg/L (ZEN)	71 h	[80]
	↓, ↓, ↓	Oocytes, Cow (NR)	0.3 to 30 μg/ml (ZEN, α-ZOL, ZAN)	24 h	[79]
**Epigenetic Modifications**	↑	Oocytes, Mouse (ICR)	10 to 50 μM (ZEN)	12 h	[103]
	↑	Oocytes, Pig (NR)	5 to 30 μM (ZEN)	44 h	[82]
**Apoptosis or Markers of Apoptosis**	↑	Granular KK-1 Cells, Mouse (NR)	20 μM (ZEN)	24 h	[73]
	↑	Ovaries, Mouse (CD-1)	10 to 30 μM (ZEN)	72 h	[70]
	↑	Granulosa Cells, Mouse (Kunming White)	15 to 150 μM (ZEN)	24 h	[72]
	↑	Granulosa Cells, Pig (NR)	60 to 120 μM (ZEN)	24 h	[74]
	↑	Oocytes, Pig (NR)	5 to 30 μM (ZEN)	44 h	[82]
	↑	Granulosa Cells, Pig (NR)	5 to 30 μM (ZEN)	48 h	[104]
	↑	Granulosa Cells, Pig (NR)	10 to 30 uM (ZEN)	48 h	[76]
	↓	Granulosa Cells, Cow (NR)	5 to 200 uM (β-ZOL)	24 h	[75]
	↑, ↑, ↑	Granulosa Cells, Horse (NR)	1 × 10^−7^ to 0.1 μM (ZEN, α-ZOL, β-ZOL)	72 h	[86]
**Cell Proliferation**	↓	Granulosa Cell, Mouse (ICR)	10 to 50 μM (ZEN)	24 h	[102]
	↓	Granulosa Cells, Pig (NR)	60 to 120 μM (ZEN)	24 h	[74]
	↑	Granulosa Cells, Cow (NR)	0.09 to 3.1 μM (α-ZOL)	48 h	[85]
	↓	Granulosa Cells, Cow (NR)	5 to 200 μM (β-ZOL)	24 h	[75]
	↑, NC, NC	Granulosa Cells, Horse (NR)	1 × 10^−7^ to 0.1 μM (ZEN, α-ZOL, β-ZOL)	72 h	[86]

*: (statistically significant (*p* < 0.05) increase: ↑; or decrease: ↓; ↑↓: results differed by dose, timing, exposure or tissue collection; NC: no change). Abbreviations: α-ZOL: alpha-zearalenol; GD: gestation day; IG: intragastric; IM: intramuscular; IP: intraperitoneal; NR: not reported; PND: post-natal day; PO: per os; SQ: subcutaneous; ZEN: zearalenone; ZER: zeranol.

**Table 4:** Primary outcomes in the uterus following mycoestrogen exposure.

In Vivo Studies					
**Outcome**	Impact * (↑, ↓, ↑↓, NC)	Experimental Model (Strain)	Dose (Compound, Route)	Age (Duration)	[Ref]
**Uterine Weight**	↑	Mouse (B6C3F1)	25 or 35 mg/kg (ZEN, diet)	2.5 wks (6 days)	[109]
	↑	Mouse (CD-1)	10^−2^ to 10^6^ μg/kg (ZEN, α-ZOL, SQ)	2.5 wks (3 days)	[18]
	↑	Mouse (B6C3F1)	35 mg/kg (ZEN, diet)	2.5 wks (7 days)	[110]
	↑	Mouse (ICR, ovariectomized)	0.5 to 1000 ng/kg (ZEN, a-ZOL, SQ)	6 wks (3 days)	[15]
	↑	Mouse (BALB/C)	10 mg/kg (ZEN, IG)	3 wks (14 days)	[27]
	↑	Rat (Sprague-Dawley)	0.03 to 10 μg/kg (ZEN, PO)	PND 21–24	[111]
	↓	Rat (Sprague Dawley, ovariectomized)	1 mg/kg (a-ZOL, IM)	4 wks (28 days)	[61]
	↑	Rat (Sprague Dawley, ovariectomized)	0.2 to 2 mg/kg (ZEN, SQ)	NR (3 days)	[112]
	↑	Rat (Sprague-Dawley)	0.03 to 10 μg/kg (ZEN, PO)	PND 21–24	[111]
	↑	Rat (Sprague-Dawley)	0.5 to 3.6 mg/kg (ZEN, diet)	3 wks (28 days)	[62]
	↑	Rat (Sprague-Dawley)	6 mg/kg (ZEN, diet)	3 wks (28 days)	[88]
	↑	Pig (Danbred)	0.75 mg/kg (ZEN, diet)	4 wks (21 days)	[89]
	↑	Pig (Duroc × Landrace × Large White × Pietrain)	1.5 mg/kg (ZEN, diet)	4 wks (35 days)	[17]
	↑	Pig (Duroc × Landrace × Large White)	0.5 to 1.5 mg/kg (ZEN, diet)	5 wks (35 days)	[113]
	↑	Pig (Large White × Landrace × Pietrain)	0.8 mg/kg (ZEN, diet)	7 wks (26 days)	[88]
**Pathological or Morphological Changes in Uterus**	↑	Mouse (CD-1)	10^−2^ to 10^6^ μg/kg (ZEN, α-ZOL, SQ)	2.5 wks (3 days)	[18]
	↑	Mouse (BALB/C)	10 mg/kg (ZEN, IG)	3 wks (14 days)	[27]
	↑	Mouse (Parkes)	2.5 mg/kg (ZEN, IP)	8 wks (up to 90 days)	[28]
	↑	Rat (Sprague-Dawley)	5 to 20 mg/kg (ZEN, diet)	GD 0–20	[68]
	NC	Rat (Sprague-Dawley)	0.1 to 10 mg/kg (ZER, SQ)	PND 15–19	[93]
	↑	Rat (Sprague-Dawley)	0.2 to 5 mg/kg (ZEN, IG)	PND 15–19	[92]
	↑	Rat (Sprague-Dawley)	0.03 to 10 μg/kg (ZEN, PO)	PND 21–24	[111]
	↑	Rat (Sprague-Dawley)	0.2 to 5 mg/kg (ZEN, IG)	PND 15–19	[92]
	↑	Dog (NR)	50 to 75 μg/kg (ZEN, capsule PO)	10 wks (42 days)	[114]
	↑	Pig(Danbred)	0.75 mg/kg (ZEN, diet)	4 wks (21 days)	[89]
	↑	Pig (Duroc × Landrace × Large White)	1 mg/kg (ZEN, diet)	4 wks (35 days)	[56]
	↑	Pig (Duroc × Landrace × Large White)	0.5 to 1.5 mg/kg (ZEN, diet)	5 wks (35 days)	[113]
	↑	Pig (Duroc × Landrace × Large White)	0.5 to 1.5 mg/kg (ZEN, diet)	5 wks (35 days)	[115]
**Estradiol-Binding Sites in Uterine Cytosol**	↓	Rat (Sprague-Dawley)	2.5 mg (ZEN, IG)	3 mos (5 days)	[16]
**Irregular Estrus Cycling**	↑	Mouse (CD-1)	0.5 or 10 mg/kg (ZEN, SQ)	GD 15–19	[116]
	↑	Mouse (C57BL/6J)	0.002 to 40 ppm (ZEN, PO)	GD 0.5–4.5	[117]
	↑	Mouse (CD-1)	10 mg/kg (ZEN and ZER, SQ)	2 wks (up to 24 wks	[90]
	↑	Mouse (BALB/C)	0.2 to 2 mg/kg (ZEN, SQ)	PND 1–5	[25]
	↑	Rat (Sprague-Dawley)	0.1 to 10 mg/kg (ZER, SQ)	PND 15–19	[93]
	↑	Rat (Sprague-Dawley)	0.2 and 10 mg/kg (ZEN, SQ)	PND 15–19	[87]
	↑	Rat (Sprague-Dawley)	0.2, 1, 5 mg/kg (ZEN, IG)	PND 15–19	[92]
	NC	Rat (Sprague-Dawley)	0.5 to 3.6 mg/kg (ZEN, diet)	3 wks (28 days)	[62]
**In Vitro Studies**					
**Outcome**	Impact * **(↑, ↓, ↑↓, NC)**	**Cell Type, Model (Strain)**	**Concentration (Compound)**	**Exposure Time**	**[Ref]**
**Cell Viability**	↓	Endometrial Stromal Cells, Mouse (NR)	25 to 125 μM (ZEN)	6 to 48 h	[118]
	NC, NC	Granulosa Cells, Pig (NR)	7.5 to 30 μM (α-ZOL, β-ZOL)	24 or 48 h	[101]
	NC, ↓	Endometrial Cells, Pig (Landrace)	7.5 to 30 μM (α-ZOL, β-ZOL)	24 h	[119]
**Apoptosis or Markers of Apoptosis**	↑	Endometrial Stromal cells, Mouse (NR)	25 to 125 μM (ZEN)	24 h	[120]
	↑	Endometrial Cells, Mouse (Strain NR)	25 to 125 μM (ZEN)	6 to 48 h	[118]
**Cell Proliferation**	↓	Granulosa Cells, Pig (NR)	7.5 to 30 μM (α-ZOL, β-ZOL)	24 or 48 h	[101]
	NC, ↓	Endometrial Cells, Pig (Landrace)	7.5 to 30 μM (α-ZOL, β-ZOL)	24 h	[119]
**Myometrial Contractility**	↑↓	Uterine smooth muscle, ex vivo	10^−11^ to 10^−6^ M (ZEN, α-ZOL, β-ZOL)	3 min	[121]

*: (statistically significant (*p* < 0.05) increase: ↑; or decrease: ↓; ↑↓: results differed by dose, timing, exposure or tissue collection; NC: no change). Abbreviations: α-ZOL: alpha-zearalenol; GD: gestation day; IG: intragastric; IM: intramuscular; IP: intraperitoneal; NR: not reported; PND: post-natal day; PO: per os; SQ: subcutaneous; ZEN: zearalenone; ZER: zeranol.

**Table 5:** Primary pregnancy and fetal outcomes following mycoestrogen exposure.

In Vivo Studies					
**Outcome**	Impact * (↑, ↓, ↑↓, NC)	Experimental Model (Strain)	Dose (Compound, Route)	Age (Duration)	[Ref]
**Placental Weight**	↓	Mouse (C57/BL6/129)	40 ppm (ZEN, diet)	8 wks (GD 5.5–13.5)	[29]
	NC	Mouse (Slc:ICR)	2 to 8 mg/kg (ZEN, SQ)	8 wks (GD 1–5)	[131]
	↓	Rat (Sprague-Dawley)	5 to 20 mg/kg (ZEN, IG)	60 days (GD 7–14)	[69]
**Placental Thickness**	↓	Rat (Sprague-Dawley)	5 to 20 mg/kg (ZEN, IG)	60 days (GD 7–14)	[69]
**Placental Hemorrhage**	↑	Mouse (C57/BL6/129)	40 ppm (ZEN, diet)	2 mos GD 5.5–13.5	[29]
**Implantation Sites**	↓	Mouse (C57BL/6J)	0.002 to 40 ppm (ZEN, diet)	3 wks (up to 5 wks)	[117]
	↓	Mouse (Slc:ICR)	2 to 8 mg/kg (ZEN, SQ)	8 wks (GD 1–5)	[131]
	↓	Rat (Sprague-Dawley)	0.3 to 146 mg/kg (ZEN, diet)	9 wks (GD 0–7)	[22]
**Embryo Size**	↓	Pig (Landrace × Large White)	1 to 10 mg/kg (ZEN, diet)	NR (GD 7–14)	[132]
**Pregnancy Rate**	↓	Mouse (C57BL/6J)	0.002 to 40 ppm (ZEN, diet)	3 wks (up to 5 wks)	[117]
	↓	Mouse (C57BL/6J)	0.8 to 40 ppm (ZEN, PO)	GD1—up to 10 wks	[117]
	↓	Rat (Sprague-Dawley)	1 to 8 mg/kg (ZEN, IG)	NR (GD 6–19)	[67]
	NC	Cow (Charolais × Balancer)	36 mg (ZER, implant)	8 mos (195 days)	[105]
**Gestational Weight Gain**	↓	Mouse (ICR)	1 to 100 mg/kg (ZER, IG)	8 wks (GD 13.5–16.5)	[66]
	↓	Mouse (Albino)	25 mg/kg (ZEN, IG)	10 wks (GD 6–13)	[133]
	↓	Rat (Sprague-Dawley)	0.3 to 146 mg/kg (ZEN, diet)	9 wks (GD 0–7)	[22]
	↓	Rat (Sprague-Dawley)	1 to 8 mg/kg (ZEN, PO)	NR (GD 6–19)	[67]
	↓	Rat (Sprague-Dawley)	5 to 20 mg/kg (ZEN, PO)	NR, GD 0–20	[68]
**Resorbed Fetuses and Fetal Deaths**	↑	Mouse (ICR)	1 to 100 mg/kg (ZER, IG)	8 wks (GD 13.5–16.5)	[66]
	↑	Mouse (Albino)	25 mg/kg (ZEN, IG)	10 wks (GD 6–13)	[133]
	↑	Rat (Sprague-Dawley)	1 to 8 mg/kg (ZEN, PO)	NR (GD 6–19)	[67]
	↑	Rat (Sprague-Dawley)	0.3 to 146 mg/kg (ZEN, diet)	9 wks (GD 0–7)	[22]
**Number of Live Births**	↓	Mouse (Slc:ICR)	2 to 8 mg/kg (ZEN, SQ)	8 wks (GD 1–5)	[131]
	↓	Mouse (Albino)	25 mg/kg (ZEN, IG)	10 wks (GD 6–13)	[133]
	↓	Mouse (ICR)	1 to 100 mg/kg (ZER, IG)	8 wks (GD 13.5–16.5)	[66]
	↓	Rat (Sprague-Dawley)	5 to 20 mg/kg (ZEN, diet)	NR, GD 0–20	[68]
	↓	Rat (Sprague-Dawley)	0.3 to 146 mg/kg (ZEN, diet)	9 wks (GD 0–7)	[22]
	↓	Pig (crossbred)	36 mg (ZER, implant)	5.5 mos old (58 days)	[134]
**Fetal Weight**	↓	Mouse (Albino)	25 mg/kg (ZEN, IG)	10 wks (GD 6–13)	[133]
	↓	Mouse (Slc:ICR)	2 to 8 mg/kg (ZEN, SQ)	8 wks (GD 1–5)	[131]
	↓	Mouse (Albino)	25 mg/kg (ZEN, IG)	10 wks (GD 6–13)	[133]
	↓	Rat (Sprague-Dawley)	1 to 8 mg/kg (ZEN, IG)	NR (GD 6–19)	[67]
	↓	Rat (Sprague-Dawley)	0.3 to 146 mg/kg (ZEN, diet)	9 wks (GD 0–7)	[22]
	↓	Pig (crossbred)	36 mg (ZER, implant)	5.5 mos old (58 days)	[134]
	↓	Rat (Sprague-Dawley)	5 to 20 mg/kg (ZEN, IG)	60 days (GD 7–14)	[69]
**Fetal Length**	↓	Rat (Sprague-Dawley)	1 to 8 mg/kg (ZEN, IG)	NR (GD 6–19)	[67]
	↓	Rat (Sprague-Dawley)	0.3 to 146 mg/kg (ZEN, diet)	9 wks (GD 0–7)	[22]
	↓	Mouse (Albino)	25 mg/kg (ZEN, IG)	10 wks (GD 6–13)	[133]
	↓	Pig (crossbred)	36 mg (ZER, implant)	5.5 mos old (58 days)	[134]
**Fetal Skeletal Abnormalities**	↑	Rat (Sprague-Dawley)	1 to 8 mg/kg (ZEN, IG)	NR (GD 6–19)	[67]
	↑	Mouse (Albino)	25 mg/kg (ZEN, IG)	10 wks (GD 6–13)	[133]
**In Vitro Studies**					
**Outcome**	Impact * **(↑, ↓, ↑↓, NC)**	**Cell Type, Model**	**Concentration (Compound)** **μM (α-ZOL, β-ZOL)**	**Exposure Time**	**[Ref]**
**Fertilization Rate**	↑↓	Porcine oocytes	1 to 1000 μg/L (ZEN)	During fertilization	[80]
**Blastocyst Formation and Development**	↓	Porcine zygotes	3.75 to 30 μM (α-ZOL)	5 days	[81]
	↓	COCs	0.312–31.2 μmol/L (ZEN, α-ZOL β-ZOL)	44 h	[135]
	↓	Fertilized porcine embryos	3 to 60 μM (α-ZOL)	24–48 h post-insemination	[136]
	↓	Bovine oocytes	3 to 30 μM (α-ZOL β-ZOL)	During in vitro maturation	[137]
	↓	Porcine blastocysts	5 to 50 μM (ZEN)	24 h	[138]
	↓	Porcine embryos	10 μM (ZEN)	144 h	[139]
**Embryotoxicity**	↑	mESC	2–20 μg/mL (ZEN)	24 h	[140]
	↑	hESC	2–20 μg/mL (ZEN)	24 h	[140]
**Corticotropic-Releasing Hormone**	↑	JEG-3 cells	0.01 to 100 nM (ZER)	24 h	[129]
**Human Chorionic Gonadotropin**	↑	BeWo cells	0.1 to 200 μM (ZEN)	24 to 72 h	[127]
	↑, NC, NC	BeWo cells	0.1 to 100 μM (ZEN, α-ZOL, β-ZOL)	48 h	[128]
**Aromatase**	↓	JEG-3 cells	0.001 to 100 μM (ZEN, ZER)	24 h	[141]
**Transient Receptor Potential Channels**	↑↓	JEG-3 cells	0.01 to 100 nM (ZER)	[130]	24 h

*: (statistically significant (*p* < 0.05) increase: ↑; or decrease: ↓; ↑↓: results differed by dose, timing, exposure or tissue collection; NC: no change). Abbreviations: α-ZOL: alpha-zearalenol; β-ZOL: beta-zearalenol; COCs: cumulus-oocyte complexes; GD: gestation day; hESC: human embryonic stem cell; IG: intragastric; IM: intramuscular; IP: intraperitoneal; mESC: mouse embryonic stem cell; NR: not reported; PND: post-natal day; PO: per os; SQ: subcutaneous; ZAN: zearalanone; ZEN: zearalenone; ZER: zeranol.

## Data Availability

Not applicable.

## Supplementary Materials

The following are available online at https://www.mdpi.com/article/10.3390/toxins13060373/s1: Table S1. Database Search Strategy, Table S2. ToxR Scores for full text papers reviewed, Table S3. Impact of mycoestrogens on circulating hormones in vivo and in vitro, Table S4. Impact of mycoestrogens on the ovary in vivo and in vitro, Table S5. Impacts of mycoestrogens on the uterus, in vivo and in vitro, Table S6. Impact of mycoestrogens on the placenta in vivo and in vitro, Table S7. Impact of Mycoestrogens on Fertilization, Pregnancy, and Fetal Development in vitro and in vivo, Table S8. PRISMA Checklist.
