# Supplementary material for: Impact of Fusarium-Derived Mycoestrogens on Female Reproduction: A Systematic Review
Source: Toxins (Basel). 2021 May 24;13(6):373. doi: 10.3390/toxins13060373 (PMC8225184; doi:10.3390/toxins13060373)
Supplement: Supplementary file 1 [file toxins-13-00373-s001.zip › toxins-1222987-supp after proof.pdf]

Impact of Fusarium-Derived Mycoestrogens on Female Reproduction: A Systematic Review

Carolyn W. Kinkade, Zorimar Rivera-Núñez, Ludwik Gorcyzca, Lauren M. Aleksunes and Emily S. Barrett

Table S1. Database Search Strategy.

Database	Search Strategy
Database: PubMed (Medline) Date of Initial Search: 11/22/19 Number of Articles: 1460	Search ((((((((((((((((((((((((((((((((((((“Extravillous trophoblasts”) OR “Labyrinth Zone”) OR gcma) OR Gcm1) OR “Syncytin 2”) OR “Syncytin 1”) OR “trophoblasts”[MeSH Terms]) OR syncytiotrophoblasts) OR spongiotrophoblast) OR “Giant trophoblasts”) OR “Junctional Zone”) OR “uterus”[MeSH Terms]) OR utero) OR “reproduction”[MeSH Terms]) OR reproduct*) OR antenatal) OR prenatal) OR “birth weight”) OR “mothers”[MeSH Terms]) OR maternal) OR “fetus”[MeSH Terms]) OR fetal) OR “embryonic structures”[MeSH Terms]) OR embryo) OR embryo*) OR “placenta”[MeSH Terms]) OR placenta) OR placenta*) OR gestation*) OR gestation) OR “premature birth”) OR pregnancy)) OR ((((((((“infertility”[MeSH Terms]) OR sterility) OR “Sperm count”) OR “Semen quality”) OR fecundity) OR fertility) OR Fertil*))) OR ((((((((((((((((((development) OR “menarche”[MeSH Terms]) OR menarche) OR “breast”[MeSH Terms]) OR breast) OR “vaginal opening”) OR “uterus”[MeSH Terms]) OR uterus) OR follicles) OR “ovary”[MeSH Terms]) OR ovaries) OR “testis”[MeSH Terms]) OR testes) OR thelarche) OR “puberty”[MeSH Terms]) OR puberty) OR “mammary glands, human”[MeSH Terms]) OR mammary) OR prepubertal))) OR ((development) OR “menarche”[MeSH Terms]) OR menarche) OR “breast”[MeSH Terms]) OR breast) OR “vaginal opening”) OR “uterus”[MeSH Terms]) OR uterus) OR follicles) OR “ovary”[MeSH Terms]) OR ovaries) OR “testis”[MeSH Terms]) OR testes) OR thelarche) OR “puberty”[MeSH Terms]) OR puberty) OR “mammary glands, human”[MeSH Terms]) OR mammary) OR prepubertal)) OR steroidogenesis) OR “sex steroid”) OR “androgens”[MeSH Terms]) OR androgen) OR androgen*) OR “pituitary gland”) OR “follicle stimulating hormone”) OR “luteinizing hormone”) OR “corticotropin-releasing hormone”) OR hyperestrogenism) OR “testosterone”[MeSH Terms]) OR testosterone) OR “estradiol”[MeSH Terms]) OR estradiol) OR “progesterone”[MeSH Terms]) OR progesterone) OR “estrogens”[MeSH Terms]) OR estrogen) OR Estrogen*) OR “Hormone balance”) OR “hormones”[MeSH Terms]) OR hormone) OR hormon*) OR “endocrine system”[MeSH Terms]) OR endocrine) OR “endorcrine dispruptor”) OR “endocrine disruptor”[MesH terms])))) AND ((((((((((beta zearal*) OR alpha-zearalanol) OR zearalenol) OR mycoestrogen) OR zearalenol) OR taleranol) OR zearalenone) OR “zeranol”[MeSH Terms]) OR zeranol) OR “zearalenone”[MeSH Terms]) OR zearalenone)) Field: Title/Abstract Sort by: Best Match
Database: Web of Science Date of Initial Search: 11/22/19 Number of Articles: 1560	#2 AND #1 *Indexes = SCI-EXPANDED, SSCI, A&HCI, CPCI-S, CPCI-SSH, BKCI-S, BKCI-SSH, ESCI, CCR-EXPANDED, IC Timespan = All years*<br><br>#2(TS = (pregnancy OR premature OR gestation OR placenta OR placental OR embryo OR fetal OR maternal OR birth OR weight OR prenatal OR antenatal OR reproductive OR perinatal OR utero OR endocrine OR hormone OR hormone AND balance OR estrogen OR progesterone OR estradiol OR testosterone OR hyperestrogenism OR corticotropin-releasing OR luteinizing OR follicle-stimulating OR pituitary OR androgen OR steroid OR steroidogenesis OR fertility OR fecundity OR semenor AND sperm OR sterility OR infertile OR prepubertal OR mammary OR puberty OR thelarche OR testes OR ovaries OR follicles OR uterus OR vaginal AND opening OR breast OR menarche OR development)) *AND* **LANGUAGE:** (English) *AND* **DOCUMENT TYPES:** (Article) *Indexes = SCI-EXPANDED, SSCI, A&HCI, CPCI-S, CPCI-SSH, BKCI-S, BKCI-SSH, ESCI, CCR-EXPANDED, IC Timespan = All years*<br><br>#1(TS = (zearalanone OR zearalenone OR alpha-zearalenol OR beta-zearalenol OR mycoestrogen OR zeranol OR zearalenols OR taleranol)) *AND* **LANGUAGE:** (English) *AND* **DOCUMENT TYPES:** (Article)

	Indexes = SCI-EXPANDED, SSCI, A&HCI, CPCI-S, CPCI-SSH, BKCI-S, BKCI-SSH, ESCI, CCR-EXPANDED, IC Timespan = All years
Database: Scopus<br>Date of Initial Search: 11/22/19<br>Number of Articles: 1633	Pregnancy OR premature birth OR gestation OR placenta OR placental OR embryo OR fetal OR maternal OR birth weight OR prenatal OR antenatal OR reproductive OR perinatal OR utero OR Junctional Zone OR Giant trophoblasts OR Spongiotrophoblasts OR Syncytiotrophoblasts OR Syncytin 1 OR Syncytin 2 OR Gcm1 OR Gcma OR Labyrinth Zone OR Extravillous trophoblasts OR Endocrine disruptor OR endocrine OR hormone OR Hormone balance OR Estrogen OR Progesterone OR Estradiol OR testosterone OR hyperestrogenism OR corticotropin-releasing hormone OR luteinizing hormone OR follicle stimulating hormone OR pituitary gland OR androgen OR sex steroid OR steroidogenesis OR Fertility OR Fecundity OR Semen quality OR Sperm count OR Sterility OR Infertile OR Prepubertal OR mammary OR puberty OR thelarche OR testes OR ovaries OR follicles OR uterus OR vaginal opening OR breast OR menarche OR development

Table S2. ToxR Scores for full text papers reviewed.

Klimish Score	Author, Year	Klimish Score	Author, Year	Klimish Score	Author, Year
1	Abbasian, N., 2018	1	Pan, P., 2020	1	Zhao, F., 2014
1	Afriyie-Gyawu, E., 2007	1	Parandin, R., 2017	1	Zhou, M. and Yang, L., 2018
1	Ahmad, B., 2018	1	Pizzo, F., 2015	1	Zhou, M., 2018
1	Althali, N., 2019	1	Pizzo, F., 2016	1	Zhou, M., 2019
1	Bernhoft, A., 2001	1	Prouillac, C., 2009	1	Zhu, C., 2014
1	Cao, H., 2019	1	Prouillac, C., 2012	1	Zhu, L., 2012
1	Chen, F., 2019	1	Ranzenigo, G., 2008	1	Zhu, Y., 2016
1	Chen, X., 2015	1	Rykaczewska, A., 2019	1	Zielonka, L., 2020
1	Collins, T., 2006	1	Sambuu, R., 2011	1	Zwierzchowski, W., 2005
1	Dai, M., 2016	1	Schoevers, E., 2012	1	Zwierzchowski, W., 2006
1	Delfosse, V., 2015	1	Seyed Toutounchi, N., 2019	2	Alm, H., 2002
1	Dellafiora, L., 2017	1	Szilagyi, J., 2017	2	Altavilla, D., 2001
1	Denli, M., 2015	1	Szilagyi, J., 2019	2	Devine T., et al., 2015
1	Denli, M., 2017	1	Takemura, H., 2007	2	Gao, X., 2017
1	Frizzell, C., 2011	1	Tan, S., 2020	2	Gao, X., 2018
1	Gajecka, M., 2011	1	Tatay, E. , 2014	2	He, J., 2018
1	Giammarino, A., 2008	1	Teixeira, L., 2011	2	Hu, J., 2016
1	Han, J., 2015	1	Tiemann, U. and Tomek, W.,	2	Jakimiuk, E., 2010
1	Heneweer, M., 2007	1	Trout, W., 2007	2	Jakimiuk, E., 2010
1	Hou, Y., 2015	1	Turcotte, J. C., 2005	2	Jiang, S., 2010
1	Jefferson, W., 2002	1	Wang, H. , 2018	2	Nazar, M., 2017
1	Kriszt, R., 2015	1	Wang, Y. , 2013	2	Nikaido, Y., 2003
1	Kunishige, K., 2017	1	Wang, Y. , 2014	2	Samik, A., 2017
1	Lai, F., 2018	1	Wang, Y., 2013	2	Silva, T., 2019
1	Lemke, S., 2001	1	Wang, Y., 2018	2	Song, T., 2020
1	Li, R., 2019	1	Warth, B., 2019	2	Stopa, E., 2019
1	Li, Y., 2015	1	Wollenhaupt, K., 2004	2	Tian, Y., 2020
1	Liu, K., 2017	1	Wu, F., 2020	2	Tiemann, U. and Viergutz, T., 2003
1	Malekinejad, H., 2007	1	Wu, L., 2020	2	Wang, H., 2012
1	Matthews, J., 2002	1	Xie, H., 2016	2	Xu, Y., 2019
1	Minervini, F., 2001	1	Xie, H., 2019	2	Yang, F., 2019
1	Minervini, F., 2006	1	Yang, L., 2018	2	Zhang, F., 2019
1	Mitak, M., 2002	1	Yang, R., 2016	2	Zhang, G., 2017
1	Nakamura, U., Kadokawa, H., 2015	1	Yao, S., 2021	2	Zhang, G., 2018
1	Nakamura, U., Rudolf, F., 2015	1	Yao, X., 2020	3	Dai, S., 2004
1	Nikaido, Y., 2004	1	Yi, Y., 2020	3	Wasowicz, K., 2005
1	Nikaido, Y., 2005	1	Yuri, T., 2004	3	Reiter, M., 2007
1	Obremski, K., 2003	1	Zhang, G., 2017	3	Lu, Y., 2018
1	Oliver, W., 2012	1	Zhang, Y., 2014		
		1	Zhao, F., 2013		

Table S3. Impact of mycoestrogens on Circulating Hormones in vivo and in vitro.

Author, Year	Species, Strain, Cell Type	Compounds Studied	Dose (route)	Timing of Exposure	Main Findings
**In Vitro Studies**					
**Pig**					
He et al., 2017	AP cells	ZEN, α-ZOL	5 to 50 µM	24 hours	• ZEN and α-ZOL decreased FSH synthesis and secretion<br>• No change in LH synthesis and secretion<br>• GPR30 expressed at higher levels than ERα and ERß
**Cow**					
Rudolf et al., 2015	AP cells	ZER	0.001 to 100 nM		• Pre-treatment with ZER (0.001-1 nM) inhibited GnRH-stimulated LH secretion<br>• Zeranol decreased cAMP, but not LHα, LHß, or FSHß
Nakamura et al., 2015	AP Cells	ZEN, α- and ß- ZOL, α- and ß- ZAL	0.001 to 10 nM	5 minute pre-treatment	• GnRH-stimulated LH secretion was inhibited by pretreatment with ZEN, α-ZAL, ZAN, ß-ZAL, α-ZOL, ß-ZOL<br>• Pretreatment with 0.01 nM of ZEN analogs in the presence of G36 had no suppressive effect on GnRH-stimulated LH secretion.<br>• ZEN analog–induced suppression of LH secretion was inhibited by pretreatment with a GPR30 antagonist
**In Vivo Studies**					
**Mouse**					
Wang, Li et al., 2013	ICR	ZER	1 to 100 mg/kg (PO)	• N = N/A<br>• Age: 6-8 week old<br>• Treatment Duration: GD 13.5-16.5	• At 100 mg/kg, plasma P_4 is significantly increased<br>• ZER reduces plasma T at all tested concentrations<br>• E_2 was unchanged by treatment<br>• ZER increases plasma P_4 at 100 mg/kg dose level
Parandin et al., 2017	BALB/C	ZEN	0.2 to 2 mg/kg (SQ)	• N = 11-12<br>• Age: Neonatal<br>• Treatment Duration: PND 1-5	• ZEN decreased LH levels and increased E_2 plasma levels

Wang, Zhang et al., 2018	BALB/C	ZEN	10 mg/kg (IG)	• N = 10<br>• Age: 3 week old<br>• Treatment Duration: 2 weeks	• ZEN increased LH levels and decreased E_2 serum levels
Ahmad et al., 2018	Parkes	ZEN	2.5 mg/kg (IP)	• N = 10<br>• Age: 8 week old<br>• Treatment Duration: Up to 90 days	• ZEN decreased LH, FSH, E_2 and P_4
Tan et al., 2020	CD1	ZEN	20 to 40 µg/kg (PO)	• N = 20<br>• Age: 4 week old<br>• Treatment Duration: 2 weeks	• ZEN decreased serum AMH, E_2, FSH, and LH
**Rat**					
Altavilla et al., 2001	Sprague-Dawley	α-ZOL	1 mg/kg (IM)	• N = 6<br>• Age: N/A<br>• Treatment Duration: 4 weeks	• Plasma E_2
Collins et al., 2006	Sprague-Dawley	ZEN	1 to 8 mg/kg (PO)	• N = 27<br>• Age: N/A<br>• Treatment Duration: GD 6-19	• No change in LH, reduction in FSH (but higher at 8 mg/kg)<br>• PRO only higher at 8, E_2 decreased at 2, 4, 8 mg/kg<br>• Conclude NOAEL less than 1 mg/kg
Zhang et al., 2014	Sprague-Dawley	ZEN	0.3 to 146.0 mg/kg (PO)	• N = N/A<br>• Age: N/A<br>Treatment Duration: GD 0-7	• Higher ZEN dose associated with lower maternal E_2 and P_4 (but higher FSH and prolactin on GD20)<br>• Decrease in P_4 and E_2 in all treatment groups

Denli et al., 2016	Sprague-Dawley	ZEN	0.5 to 3.6 mg/kg (PO)	• N = 5<br>• Age: 3 week old<br>• Treatment Duration:<br>• 4 weeks	• FSH was not changed by ZEN
Abbasian et al., 2018	Wistar albino	ZEN	0.1 and 1 mg/kg (PO)	• N = 8<br>• Age: 9-10 week old<br>• Treatment Duration: 3 months	• P_4, insulin, and glucose increased in a dose- and time-dependent manner in response to ZEN<br>• The levels of T and LH increased significantly in ZEN-treated rats in comparison to the control group<br>• The level of E_2 and FSH decreased significantly in ZEN group as compared to the control rats<br>• The level of TNF-α was increased significantly in the ZEN-treated group as compared to the control group<br>• The expression of Sfrp4 was significantly up-regulated in the ZEN group in comparison with the control rats
Pan et al., 2020	Sprague-Dawley	ZEN	2.5 to 20 mg/kg	• N = 6<br>• Age: NR<br>• Treatment Duration: GD 14-21	• ZEN decreased LH and FSH at 5 to 20 mg/kg
**Pig**					
Chen et al., 2015	Landrace x Yorkshire x Duroc	ZEN	1.1 to 3.2 mg/kg (PO)	• N = 5<br>• Age: 2 week old<br>• Treatment Duration: 18 days	• Linear change in T, P_4, E_2, LH, FSH but not PRO<br>• Increase in PRO significant<br>• Decrease (<0.05) of LH in the highest dose treatment group.
He et al., 2017	Cross-bred	ZEN, α-ZOL	7.54 mg/kg (IP)	• N = 8<br>• Age: N/A<br>• Treatment Duration: 24 hours	• ZEN and α-ZOL decreased FSH synthesis and secretion but not in LH<br>• GPR30 expressed at higher levels than ERα and ERß. This may occurred by the transcription factor LHX3 (was involved in the mechanism of ZEN and α-ZOL actions on gonadotropes in the pituitary)
Rykaczewska et al., 2019	Pigs	ZEN	5 to 15 µg/kg (PO)	• N = 10-15<br>• Age: Prepubertal<br>• Treatment Duration: 42 days	• ZEN increased E_2, while decreasing P_4 and T levels

Zhou et al., 2019	Duroc x Landrace x Large White	ZEN	1 mg/kg (PO)	• N = 10<br>• Age: 4 week old<br>• Treatment Duration: 35 days	• Serum LH in ZEN was lower than control but higher than estradiol benzoate<br>• Higher FSH and P_4 in ZEN and control than estradiol benzoate<br>• Serum glutathione peroxidase in ZEN was lower than control and estradiol benzoate<br>• Malondiadehyde in the ZEN group was higher than control or estradiol benzoate group
Zielonka et al., 2020	NR	ZEN	20 to 40 µg/kg (PO)	• N=6<br>• Age: pre-pubertal<br>• Treatment Duration: 48 days	• Plasma E_2 increased between days 36 and 42<br>• Plasma T fluctuated during ZEN exposure
Wu, F., et al, 2021	Landrace x Yorkshire	ZEN	200 to 1600 µg/kg (PO)	• N = 12<br>• Age: Pre-pubertal<br>• Treatment Duration: 14 days	• Serum LH and E_2 were significantly reduced

Abbreviations: α-ZOL: alpha-zearalenol; E_2: estradiol; FSH; follicle stimulating hormone; GD: gestation day; IG: intragastric; IM: intramuscular; IP: intraperitoneal; LH: luteinizing hormone; NR: Not reported; PND: post-natal day; PO: per os; P_4: progesterone; PRO: prolactin; SQ: subcutaneous; T: testosterone; ZEN: zearalenone.

Table S4. Impact of mycoestrogens on the ovary in vivo and in vitro.

Author, Year	Strain, Cell Type	Compounds Studied	Dose (route)	N, Timing of Exposure	Main Findings
**In Vitro Studies**					
**Mouse**					
Zhu, Hou et al., 2014	ICR, oocytes	ZEN	10 to 50 µM	12 hours	• No impact of ZEN on egg developmental competence<br>• Increased 5-methyl-cytosine level in ZEN treated eggs<br>• Increased DNA methylation level in ZEN treated eggs<br>• Decreased levels of H3K4me2, H3K9me3, and H4K20me1, me2, me3 levels in ZEN treated eggs
Hou et al., 2015	ICR, oocytes	ZEN	10 to 50 µM	24 hours	• ZEN-treated oocyte maturation rates were decreased<br>• ZEN treatment resulted in more oocytes with abnormal spindle morphologies<br>• Actin filament expression and distribution were also disrupted after ZEN treatment<br>• ZEN reduced mouse granulosa cell proliferation in a dose-dependent manner
Li, He et al., 2015	Ovarian granular KK-1 cells	ZEN	20 µM	24 hours	• ZEN<br>o Decreased cell viability<br>o Increased ROS<br>o Increased MDA/mg protein<br>• Increased Bax, Casp3, and Casp9 mRNA and protein
Zhang, Sun, et al., 2017	CD1, Ovaries *ex vivo*	ZEN	10 to 30 µM	72 hours	• Newborn pup ovaries exposed to 10 or 30 µM ZEN in vitro had significantly less germ cell numbers<br>• ZEN increased apoptosis in ovaries<br>• ZEN reduced mRNA of oocyte specific genes<br>o LIM homeobox 8 (*Lhx8*)<br>o newborn ovary homeobox (*Nobox*)<br>o spermatogenesis and oogenesis helix-loop-helix (*Sohlh2*)<br>• Changes in Lhx8 3' – UTR DNA Methylation dynamics in oocytes<br>• Severely impaired folliculogenesis in ovaries after transplantation under the kidney capsule of immunodeficient mice
Chen et al., 2018	Kunming White, ovarian granulosa cells	ZEN	15 to 150 µM	24 hours	• ZEN decreased cell viability and increased apoptosis in a dose-dependent manner<br>• ZEN upregulates LC3-11 (autophagy response)<br>• ZEN inhibits mTOR and ERK1/2 signaling pathways<br>• ZEN activated ER stress by upregulating the stress-regulated proteins GRP78, HERP, and CHOP
Yi, Y., 2020	Kunming, ovarian	ZEN	10 to 120 µM	24 hours	• ZEN reduces survival rate in a dose-dependent manner from 30 µM

	granulosa cells				
**Hamster**					
Tatay et al., 2014	CHO-K1 cells	ZEN, α- and β-ZOL	0 to 100 µM	24, 48 or 72 hours	• IC50>100 µM for ZEN<br>• IC50 33.00 for α-ZOL<br>• IC50 >75 for ß-ZOL<br>• No conversion of ZEN to α -ZOL or ß-ZOL
**Pig**					
Alm et al., 2002	Landrace, ovaries and oocytes	α- and β-ZOL	3.5 to 90 µM	48 hours	• Dose dependent impacts on maturation (α starting at 7.5, β at 30 µM)<br>• Significantly greater oocyte degeneration at 30 µM compared to controls
Tiemann, Tomek et al., 2003	Strain N/A, Granulosa cells	α- and β-ZOL	5 to 30 µM	24 hours	• ZEN did not impact cell viability
Sambuu et al., 2011	Strain N/A, oocytes	ZEN	1 to 1000 µg/L	ZEN supplementation of media during COC culture and fertilization	• Significantly lower maturation of oocytes<br>• No change in rate of degeneration or DNA fragmentation of oocytes
Zhu, Yuan et al., 2012	Strain N/A, Granulosa cells	ZEN	60 to 120 µM	24 hours	• ZEN reduced the proliferation of porcine granulosa cells in a dose dependent manner<br>• ZEN increased apoptosis and necrosis<br>○ Increased caspase 3 and 9 expression<br>• ZEN leads to loss of mitochondrial transmembrane potential
Han, 2015	Strain N/A, oocytes	ZEN	5 to 30 µM	44 hours	• ZEN increased ROS in oocytes<br>• ZEN increased rates of autophagy and apoptosis<br>• Increased expression of 5mC, H3K9me3, H3K27me3, and H3K4me2
Zhang, Song, et al., 2018	Strain N/A, Granulosa cells	ZEN	10 and 30 µM	72 hours	• ZEN altered mitosis associated and steroidogenesis genes in porcine cells<br>• ZEN induced the expression of inflammatory genes
Lai et al., 2018	Strain N/A, Granulosa cells	ZEN	3 to 30 µM	12 days	• ZEN led to platelet-activating factor (PAF) and lyso phosphatidylcholine (LPC) depletion in the GC media<br>• ZEN decreased the percentage of antrum formation
Zhang, Li., 2019	Strain NR, granulosa cells	ZEN	10 to 30 µM	48 hours	• ZEN elevated apoptosis, significantly at 30 µM after 48 hours<br>• ZEN exposure increased expression of key proteins in JAK2-STAT3 pathway

Tian et al., 2020	Strain NR, granulosa cells	ZEN	10 to 30 µM	48 hours	• ZEN elevated apoptosis in a dose dependent manner<br>• ZEN exposure affected expression of miRNAs associated with apoptosis-related pathways
**Sheep**					
Silva et al., 2019	Strain NR, ovarian pieces	ZEN	1 µmol/L	3 days	• ZEN significantly decreased the rate of granulosa cell proliferation<br>• ZEN induced DNA double-strand breaks in primordial follicles
**Cow**					
Minervini et al., 2001	Strain N/A, oocytes	ZEN, α- and β-ZOL	0.3 to 30 µg/ml	24 hours	• ZEN and α-ZOL delayed oocyte maturation and induced chromatin abnormalities
Pizzo et al., 2015	Strain N/A, Granulosa cells	α-ZOL	0.09 to 3.1 µM	48 hours	• α-ZOL increased cell growth at 0.09 and 0.31 µM doses
Nazar et al., 2017	Strain N/A, oocytes	α- and β-ZOL	3 to 30 µM	22 hours	• Decreased embryo cleavage<br>• Decreased blastocyst formation rate<br>• Decreased number of blastocysts
Yang, 2019	Strain N/A, Granulosa cells	β-ZOL	5 to 200 µM	24 hours	• ß-ZOL inhibited cell proliferation in a dose-dependent manner<br>• ß-ZOL induced apoptosis of granulosa cells
**Horse**					
Minervini et al., 2006	Strain N/A, Granulosa cells	ZEN, α- and β-ZOL	1 x 10^{-7} to 0.1 µM	72 hours	• ZEN increased cell proliferation<br>• Mycotoxins increased apoptosis
**In Vivo Studies**					
**Gestational Exposure**					
**Mouse**					
Nikaido et al., 2004	CD1	ZEN	0.5 or 10 mg/kg (SQ)	• N = 6<br>• Age: 2 week old<br>• Treatment Duration: GD 15-19	• Maternal exposure to ZEN increased the duration of estrous cycle<br>• Lack of corpora lutea and vaginal cornification was observed in the high-dose ZEN group
Zhao et al., 2013	C57BL/6J	ZEN	0.002 to 40 ppm (PO)	• N = 6-14<br>• Age: 3 week old	• Increased duration in estrous stage<br>• Absence of proestrous stage in 40 ppm ZEN group

				• Treatment Duration: GD 0.5-4.5	
Zhao, F., et al., 2014	C57BL/6J	ZEN	0.8 to 20 ppm (PO)	• N = 6-10<br>• Age: 8 wks<br>• Treatment Duration: GD1 - ~10 wks	• 20 ppm ZEN led to decrease in implantation rate, pregnancy rate and litter size
Liu et al., 2017	CD1	ZEN	20 to 40 μg/kg (SQ)	• N = 114<br>• Age: N/A<br>• Treatment Duration: GD 12.5-18.5	• ZEN decreased the percentage of diplotene stage germ cells<br>○ More cells remain at zygotene or pachytene stages<br>• mRNA expression level of meiosis related genes was reduced in ZEN group<br>• ZEN increased DNA-DSBs at the diplotene stage<br>• ZEN activates DNA damage repair genes *Rad51* and *Brca1*<br>• ZEN exposure decreased primordial follicles in newborn mouse ovaries
**Rat**					
Zhang et al., 2014	Sprague-Dawley	ZEN	0.3 to 146.0 mg/kg (PO)	• N = N/A<br>• Age: N/A<br>• Treatment Duration: GD 0-7	• Increased ovarian weight<br>• Fewer functional follicles<br>○ Fibrotic changes in ovaries
Gao et al., 2017	Sprague-Dawley	ZEN	5 to 20 mg/kg (PO)	• N = 12-16<br>• Age: N/A<br>• Treatment Duration: GD 0-20	• ZEN did not induce pathological changes in ovaries of weaned F1 rats<br>• ZEN induced follicular atresia in F1 female adult rats<br>• Protein levels of ESR1 and 3ß-hydroxysteroid dehydrogenase were decreased in the adult ovaries
**Prepubescent Exposure**					
**Mouse**					
Nikaido et al., 2005	CD1	ZEN, ZER	10 mg/kg (SQ)	• N = 17-24<br>• Age: 2 week old<br>• Treatment Duration:	• ZEN and ZER accelerated puberty onset and caused more time in estrus<br>• Absence of corpora lutea in ZEN (100%) and ZER (100%) animals at 4 weeks compared to control (33%), and 33% of ZEN group at 8 weeks

				Up to 24 weeks of age	
Parandin et al., 2017	BALB/C	ZEN	0.2 to 2 mg/kg (SQ)	• N = 11-13<br>• Age: Neo-nates<br>• Treatment Duration: 4 days	• Disrupted estrus cycles and decreased follicular profiles were observed following ZEN exposure
Wang et al., 2018	BALB/C	ZEN	10 mg/kg (IG)	• N = 10<br>• Age: 3 week old<br>• Treatment Duration: 2 weeks	• Ovaries in ZEN group<br>○ Increased number of non-functional follicles<br>○ Thickened granular layer<br>○ Uneven follicular antral fluid
**Rat**					
Nikaido et al., 2003	Sprague-Dawley	ZEN	0.2 and 10 mg/kg (SC)	• N = 30<br>• Age: 2 week old<br>• Treatment Duration: PND 15-19	• ZEN did not impact ovarian weight<br>• ZEN decreased the number of normal-cycling animals
Yuri et al., 2004	Sprague-Dawley	ZER	0.1 to 10 mg/kg (SQ)	• N = 30<br>• Age: 2 week old<br>• Treatment Duration: 4 days	• Irregular estrous cycles<br>○ Prolonged estrous<br>○ Prolonged diestrous<br>• Anovulatory ovary
Denli et al., 2015	Sprague-Dawley	ZEN	6 mg/kg (PO)	• N = 8<br>• Age: 3 week old<br>• Treatment Duration: 28 days	• ZEN increased the relative weight of ovaries
Yang et al., 2016	Sprague-Dawley	ZEN	0.2, 1 or 5 mg/kg (IG)	• N = 7	• Irregularity in estrous cyclicity

				• Age: 2 week old<br>• Treatment Duration: PND 15-19	• Advanced development of ovaries<br>• Increased number of mature follicles
Denli et al., 2017	Sprague-Dawley	ZEN	0.5 to 3.6 mg/kg (PO)	• N = 12<br>• Age: 3 week old<br>• Treatment Duration: 28 days	• ZEN did not impact ovary weight or inter-oestrous interval
Kriszt et al., 2015	Wistar	ZEN	10 mg/kg (PO)	• N=10<br>• Age: 18 days<br>• Treatment Duration: 10 days	• ZEN increased number of antral follicles in the ovary
**Pig**					
Obremski et al., 2003	Large White x Landrace	ZEN	200 to 400 µg/kg (PO)	• N = 8<br>• Age: 4 month old<br>• Treatment Duration: 7 days	• Ovarian follicle atresia<br>• Apoptosis-like changes in granulosa cells<br>• Increased cell proliferation in oviduct (77%)
Zwierzchowski et al., 2005	Large White x Landrace	ZEN	200 µg/kg (PO)	• N = 4<br>• Age: 3 month old<br>• Treatment Duration: 8 days	• ZEN decreased the number of ovarian follicles<br>• Follicles were in the cortical area
Jakimiuk et al., 2010	Strain N/A	ZEN	20 to 40 µg/kg (PO)	• N = 4<br>• Age: 2 month old<br>• Treatment Duration: 48 days	• ZEN increased the number of medium-sized ovarian follicles

Gajecka et al., 2011	Strain N/A	ZEN	20 to 40 µg/kg (PO)	• N = 4<br>• Age: 2 month old<br>• Treatment Duration: 48 days	• Long term low dose ZEN exposure<br>• ZEN lowered the proliferative ability of granulosa cells of the ovarian follicle walls and connective tissue of the ovarian stroma
Teixeira et al., 2011	Danbred	ZEN	0.75 mg/kg (PO)	• N = 6<br>• Age: 4 week old<br>• Treatment Duration: 21 days	• ZEN increased ovary weight
Schoevers et al., 2012	York x Finn-ish Landrace	ZEN	200 to 1000 µg/kg (PO)	• N = 7<br>• Age: N/A<br>• Treatment Duration: GD 0-112	• ZEN decreased the density of oogonia of F1 newborns between 0 and 21 days after birth<br>• Primordial/secondary follicles remained unchanged
Denli et al., 2015	Large White x Landrace x Pietrain	ZEN	6 mg/kg (PO)	• N = 8<br>• Age: 2 month old<br>• Treatment Duration: 26 days	• ZEN increased the relative weight of ovaries
Chen et al., 2015	Duroc x Landrace x Yorkshire	ZEN	1.1 to 3.2 mg/kg (PO)	• N = 5<br>• Age: 2 week old<br>• Treatment Duration: 18 days	• ZEN increased ovary size<br>• ZEN decreased the number of primordial follicles and primary growing follicles<br>• Antiapoptotic effects on ovarian granulosa cells
Dai et al., 2016	Duroc x Landrace x Yorkshire	ZEN	1.04 mg/kg (PO)	• N = 10<br>• Age: 4 week old<br>• Treatment Duration: 35 days	• ZEN increased the number of growing follicles and their diameter

Yang et al., 2018	Duroc x Landrace x Large White	ZEN	0.5 to 1.5 mg/kg (PO)	• N = 10<br>• Age: 4 week old<br>• Treatment Duration: 35 days	• ZEN decreased primordial follicles and increased atretic follicles
**Pubescent Exposure**					
**Mouse**					
Samik et al., 2017	Strain N/A	ZEN	0.1 mg/d (PO)	• N = N/A<br>• Age: 8-10 week old<br>• Treatment Duration: 10 days	• Significant decrease in the number of primary follicles, secondary follicles, tertiary follicles, and de Graff's follicle<br>• No change in corpus luteum
Ahmad et al., 2018	Parkes	ZEN	2.5 mg/kg (IP)	• N = 10<br>• Age: 8 week old<br>• Treatment Duration: Up to 90 days	• ZEN induced thickening, swelling, and shortening of the uterus and ovaries<br>• 30 Day ZEN treatment<br>○ Reduction number of large and mature follicles<br>○ Loosely arranged follicular cells<br>○ Atretic follicles<br>○ Lack of corpus luteum<br>• Observations at 30 days progressed after continued treatment till 90 days
Tan et al., 2020	CD1	ZEN	20 to 40 μg/kg (PO)	• N = 20<br>• Age: 4 week old<br>• Treatment Duration: 2 weeks	• ZEN decreased BW<br>• ZEN decreased number of primordial follicles
**Rat**					

Abbasian et al., 2018	Wistar albino	ZEN	0.1 and 1 mg/kg (PO)	• N = 8<br>• Age: 9-10 week old<br>• Treatment Duration: 3 months	• ZEN did not impact the morphology of ovaries
**Horse**					
Devine et al., 2016	Charolais x Balancer	ZER	36 mg (implant)	• N = 17<br>• Age: 8 month old<br>• Treatment Duration: 195 days	• ZEN altered reproductive tract score (RTS) relative to vehicle

Table S5. Impacts of mycoestrogens on the uterus, in vivo and in vitro.

Author, Year	Species, Strain, Cell Type	Compounds Studied	Dose (route)	N, Timing of Exposure	Main Findings
**In Vitro Studies**					
**Mouse**					
Hu et al., 2016	Strain N/A, Endometrial stromal cells (ESC)	ZEN	25 to 125 µM	24 hours	• ZEN increased the Bax/Bcl-2 ratio and caspase-3 and -9 protein levels in a dose-dependent manner
Xie et al., 2016	ICR, Endometrial stromal cells (ESC)	ZEN	25 to 125 µM	6 to 48 hours	• ESC viability was significantly lower at 75 µM for 6, 12, 24 hours<br>• Cell shrinkage and floating cells at 100 uM ZEN<br>• Compared with control, G2/M phase fraction was significantly higher at/or above 50 µM ZEN<br>• S phase fraction was lower at/or above 100 µM ZEN<br>• Annexin V-FITC/PI double-staining method, apoptosis rate of ESCs was significantly higher at/or above 50 µM ZEN<br>• TUNEL assay, apoptosis rate was significantly higher at/or above 75 µM<br>• Differentially expressed genes related to cell cycle, apoptosis, and embryonic development, such as<br>○ *Bec-2*<br>○ *Cdc*<br>○ *HOXa-10*
**Pig**					
Tiemann, et al., 2003	Strain N/A, Endometrial cells	α- and β-ZOL	7.5 to 30 µM	24 or 48 hours	• Viability was not affected<br>• α-ZOL did not alter cell cycle distribution<br>• β-ZOL reduced S phase and arrested G0/1 phase<br>• Decrease in PCNA with β-ZOL
Wollenhaupt et al., 2004	Landrace, Endometrial cells	α- and β-ZOL	7.5 to 30 µM	24 hours	• α-ZOL minimally altered the phosphorylation of MAP kinases, Akt, eIF4E, and 4E-BP1<br>• β-ZOL reduced phosphorylation of MAP kinases, Akt, eIF4E, and 4E-BP1<br>• 30 µM β-ZOL decreased the number of viable cells and inhibited cell proliferation
Song et al., 2020	Strain NR, porcine endometrial ells	ZEN	5 to 80 µmol/L	24 hours	• ZEN increased proportion of cells in G1 phase, decreased proportion of cells in S and G2 phase<br>• ZEN activates WNT/B-catenin pathway by up-regulating WNT1 and B-catenin expression<br>• ZEN upregulates GSK-3B and downregulates CCND1
**Sheep**					

Giam-marino et al., 2008	Uterine smooth muscle, *ex vivo*	ZEN, α- and β-ZOL	10^{-11} to 10^{-6} M	• N=20<br>• Age: 45-50 days old<br>• Treatment Duration: 3 min	• ZEN increases uterine activity similar to E_2<br>• α-ZOL inhibits myometrial contractility
**Human**					
Yao, Wei, et al., 2020	Human endometrial stromal cells	ZEN	1 to 50 µM	• 48 hours	• Significant reduction of cell viability was observed at 50 µM of ZEN 48 hours after administration<br>• ZEN exposure to reduced decidualization progress<br>• ZEN exhibited its inhibitory action through nuclear translocation of ERα
**In Vivo Studies**					
**Mouse**					
Lemke et al., 2001	B6C3F1	ZEN	25 mg/kg or 35 mg/kg (PO)	• N = 12<br>• Age: 2.5 week old<br>• Treatment Duration: 6 days	• Dose-dependent increase in uterine weight
Jefferson et al., 2002	CD-1	ZEN, α-ZOL	0.01 to 1 000 000 µg/kg (SQ)	• N = 5<br>• Age: 2.5 week old<br>• Treatment Duration: 3 days	• At 10 000 µg/kg, ZEN and α-ZOL increased uterine wet weight<br>• At/or above 10 000 µg/kg, ZEN and α-ZOL increased uterine epithelium cell height<br>• At 1000 µg/kg, ZEN increased the number of uterine glands
Afriyie-Gyawu et al., 2007	B6C3F1	ZEN	35 mg/kg (PO)	• N = 12<br>• Age: 2.5 week old<br>• Treatment Duration: 7 days	• Increased uterine:body weight ratio
Takemura et al., 2007 (ovariectomized)	ICR	ZEN, α-ZOL	0.5 to 1000 ng/kg (SQ)	• N = 10-11<br>• Age: 6-7 week old<br>• Treatment Duration: 3 days	• The IC50 values of ZEN, ZOL and E_2 for their binding to ERα were 240.4, 21.79 and 10.35 nM, respectively.<br>• The IC50 values of ZEN, ZOL and E_2 for their binding to ERß were 165.7, 42.76 and 10.04 nM, respectively.<br>• ZEN, ZOL and E_2 increased the mean uterine wet weight in ovariectomized mice in a dose-dependent manner

Ahmad et al., 2018	Parkes	ZEN	2.5 mg/kg (IP)	• N = 30<br>• Age: 8 week old<br>• Treatment Duration: Up to 90 days	• Thickening, swelling, and shortening of the uterus and ovaries
Wang et al., 2018	BALB/C	ZEN	10 mg/kg (IG)	• N = 10<br>• Age: 3 weeks old<br>• Treatment Duration: 2 weeks	• Increased wet weight<br>• Thinning myometrium<br>• Infiltrating eosinophils<br>• Fewer submucosal glands
**Rat**					
Altavilla et al., 2001	Sprague-Dawley (ovariecto-mized)	α-ZOL	1 mg/kg (IM)	• N = 6<br>• Age: N/A<br>• Treatment Duration:<br>• 4 weeks	• α-ZOL reversed endothelial dysfunction in OVX rats<br>• α-ZOL decreased uterine weight
Mitak et al., 2002	Sprague-Dawley	ZEN	2.5 mg (PO)	• N = 15<br>• Age: 3 month old<br>• Treatment Duration: 5 days	• Decreased number of receptor binding sites for E_2 in uterine cytosol
Yuri et al., 2004	Sprague-Dawley	ZER	0.1 to 10 mg/kg (SQ)	• N = 30<br>• Age: 2 week old<br>• Treatment Duration: PND 15-19	• Increased uterine-ovarian weight at 10 mg/kg ZEN
Turcotte et al., 2005	Sprague-Dawley (ovariecto-mized)	ZEN	0.2 to 2 mg (SQ)	• N = 4-7<br>• Age: N/A<br>• Treatment Duration: 3 days	• Increased uterine weight
Heneweer 2007	Sprague-Dawley	ZEN	0.03 to 10 µg/kg (PO)	• N = 4<br>• Age: 3 week old	• Increased uterine weight and epithelial cell height<br>• Degenerated epithelium at 10 µg/kg ZEN

				• Treatment Duration: PND 21-24	
Denli et al., 2015	Sprague-Dawley	ZEN	6 mg/kg (PO)	• N = 15<br>• Age: 3 week old<br>• Treatment Duration: 28 days	• Increased uterine weight
Denli et al., 2016	Sprague-Dawley	ZEN	0.5 to 3.6 mg/kg (PO)	• N = 5<br>• Age: 3 week old<br>• Treatment Duration: 4 weeks	• Increased uterine weight
Yang et al., 2016	Sprague-Dawley	ZEN	0.2 to 5 mg/kg (IG)	• N = 7<br>• Age: 2 week old<br>• Treatment Duration: PND 15-19	• Advanced development of ovaries and uterus<br>• Increased of thickness of myometrium<br>• Increased number of mature follicles
Gao et al., 2017	Sprague-Dawley	ZEN	5 to 20 mg/kg (PO)	• N = 16<br>• Age: N/A<br>• Treatment Duration:<br>• GD 0 to 21	• In F1 rats,<br>○ Thinning of the uterine layer at 20mg/kg ZEN<br>○ Fewer submucosal glands, glandular epithelial cell death and infiltrating eosinophils at 10 mg/kg ZEN<br>○ Mucosal hyperplasia and muscular layer thinning at 20 mg/kg feed ZEN<br>• Protein expression of uterine 3ß-HSD was decreased by up to 1.9-fold in 10 and 20 mg/kg ZEN groups
**Dog**					
Stopa et al., 2014	NR	ZEN	50 to 150 ug/kg (PO)	• N=10<br>• Age: 70 days<br>• Treatment Duration: 42 days	• Low doses of ZEN (100% and 150% of the NOAEL)causes simple glandular hyperplasia of the endometrium accompanied by adenogenesis, angiogenesis, and vasodilation
**Pig**					
Teixeira et al., 2011	Danbred	ZEN	0.75 mg/kg (PO)	• N = 6<br>• Age: 4 week old	• Increased weight of reproductive tract and ovaries-uterus-vagina complexes<br>• Increased proliferation of uterine epithelial cells<br>• Squamous metaplasia in uterus<br>• Hyperplasia of endometrial glands

				• Treatment Duration: 21 days	
Oliver et al., 2012	Duroc x Landrace x Large White x Pietrain	ZEN	1.5 mg/kg (PO)	• N = 10<br>• Age: 4 week old<br>• Treatment Duration: 35 days	• Increased reproductive tract weight<br>• Increased uterine endometrial gland development<br>• Increased uterine ER-β mRNA and protein level
Denli et al., 2014	Large White x Landrace x Pietrain	ZEN	0.8 mg/kg (PO)	• N = 8<br>• Age: 7 week old<br>• Treatment Duration: 26 days	• Increased uterine weight
Zhou, Yang, Shao et al., 2018	Duroc x Landrace x Large White	ZEN	0.5 to 1.5 mg/kg (PO)	• N = 10<br>• Age: 5 week old<br>• Treatment Duration: 35 days	• Increased mRNA and protein expression of uterine PCNA, BAX, BCL-2, TGF-B1, and Smad3
Zhou et al., 2018	Duroc x Landrace x Large White	ZEN	0.5 to 1.5 mg/kg (PO)	• N = 10<br>• Age: 5 week old<br>• Treatment Duration: 35 days	• Increased uterine and myometrium weight
Zhou et al., 2019	Duroc x Landrace x Large White	ZEN	1 mg/kg (PO)	• N = 10<br>• Age: 4 week old<br>• Treatment Duration: 35 days	• Increased uterine index<br>• Increased myometrium and endometrium thickness
Kriszt et al., 2015	Wistar	ZEN	10 mg/kg (PO)	• N=10<br>• Age: 18 days<br>• Treatment Duration: 10 days	• ZEN increased uterine weight

Abbreviations: α-ZOL: alpha-zearalenol; E_2: estradiol; FSH; follicle stimulating hormone; GD: gestation day; IG: intragastric; IM: intramuscular; IP: intraperitoneal; LH: luteinizing hormone; NR: Not reported; PND: post-natal day; PO: per os; P_4: progesterone**;** PRO: prolactin; SQ: subcutaneous; T: testosterone; ZEN: zearalenone.

Table S6. Impact of mycoestrogens on the placenta in vivo and in vitro.

Author, Year	Species, Strain, Cell Type	Compounds Studied	Dose (route)	Timing of Exposure	Main Findings
**Disposition in the Placenta**					
Bernhoft et al., 2001	Sprague-Dawley rats	ZEN	0.74 mg/kg (IV and IG)	• N = 3<br>• Age: N/A<br>• Treatment Duration: GD 12 and 18	• ZEN and α-ZOL found in fetus and placenta on both GD 12 and 18 at levels lower than maternal livers<br>• Concentrations of parent chemicals were higher than metabolites. Notably, placental ZEN concentrations were higher on GD12 compared 18. However, this difference was not seen in the fetus.
Lange et al., 2002	Dutch-Belted rabbits	ZER	0.25 mg/kg (SQ)	• N = 1<br>• Age: N/A<br>• Treatment Duration: GD 14	• ZER residue was observed in placentas
Szilagyi et al., 2017	BeWo cells	ZEN	10 μM	1.5 hours	• BCRP is a maternal-facing transporter that effluxes chemicals from the placenta back to the maternal circulation<br>• Disruption of cholesterol concentrations in plasma membrane reduces ZEN efflux by BCRP, which could be restored with repletion of exogenous cholesterol
Szilagyi et al., 2019	BeWo cells	ZEN	50 μM	2 hours	• In BeWo cells engineered to express reduced BCRP protein levels, an increase in the apical-to-basolateral (maternal-to-fetal) transfer of ZEN was observed.
Szilagyi et al., 2019	Wild-type and Bcrp-null C57BL/6 mice	ZEN	10 mg/kg (IV)	• N = 4-5<br>• Age: Adult<br>• Treatment Duration: GD 14	• The placentas and fetuses from Bcrp-null mice exhibited higher free and total concentrations of ZEN and α-ZOL . No difference in maternal ZEN concentrations were observed between genotypes
Warth et al., 2019	Human placentas	ZEN	1 μM	6 hours	• Perfusion of human placentas ex vivo for 6 h resulted in the transfer of ZEN, α-ZOL, and zearalenone-14-Sulf were quantified in time-dependent manner
**Effect on Placental Signaling and Health**					
**In Vitro Studies**					
**Human**					

Prouillac et al., 2009	BeWo cells	ZEN	0.1 to 200 μM	24 to 72 hours	• ZEN (5-10 μM) increased secretion of hCG secretion into media which could be blocked by ER antagonist ICI-182780 (10 μM)<br>• ZEN (10 μM) stimulated formation of multinucleated syncytial cells similar to E_2<br>• ZEN (10 μM) up-regulated MRP1 transport protein expression similar to E_2
Prouillac et al., 2012	BeWo cells	ZEN, α- and β-ZOL	0.1 to 100 μM	48 hours	• ZEN, but not α-ZOL or β-ZOL, increase hCG mRNA and secretion at 10 μM<br>• All 3 chemicals increase the mRNA expression of the cell fusion gene, *syncytin 2*. ZEN also up-regulates *syncytin 1* mRNA as well<br>• Intracellular concentrations of cyclic AMP were increased with α-ZOL and β-ZOL, but not ZEN, and may represent a mechanism responsible for increase *syncytin* mRNA expression<br>• α-ZOL and β-ZOL have differential effects on the expression of placental barrier transporters (alpha – ↑ MRP1 and 2 protein at 10 μM, slight ↓ MRP2 at 1 μM and beta – ↓ MRP1 and BCRP at 10 μM, also ↓ BCRP protein at 1 μM)<br>• Computational modeling suggested that the 3 chemicals may bind the ligand binding pocket of the progesterone receptor
Wang et al., 2013	JEG-3 cells	ZER	0.01 to 100 nM	24 hours	• ZER up-regulated the mRNA and protein expression of CRH, a hormone that regulates the onset of parturition<br>• ZER enhances binding of the CREB transcription factor to the cAMP response element (CRE) in the CRH promoter<br>• Pharmacological inhibition of the ERK pathway blocked ZER-induced CRH expression suggesting involvement of this response
Wang et al., 2014	JEG-3 cells	ZEN and ZER	ZEN (0.001 to 10 μM), ZER (0.01 to 100 nM)	24 hours	• ZEN at a concentration of 1 μM and higher reduced activity of the aromatase enzyme, but did not alter *CYP19* mRNA expression
Zhu et al., 2016	JEG-3 cells	ZER	0.01 to 100 nM	24 hours	• ZER increased TRPs, which are linked to a number of placental cell functions. ZER enhanced protein levels of TRPP2, C3, and C6 ion channels without affecting expression of TRPC4, V5, or V6<br>• ZER at concentrations of 10 nM and higher increased intracellular Ca^{2+} levels<br>• ZER up-regulated expression of the COX-2 enzyme and the apoptosis-related BCL-2 protein. Induction of *COX-2* mRNA by ZER can be blocked using a pharmacological inhibitor of the TRPC3 channel<br>• ZER enhanced signaling through numerous pathways (p-JNK, p-P38, p-ERK) that may be responsible for altering TRP signaling

Seyed Toutounchi et al., 2019	BeWo cells	ZEN	2 to 8 μM	24 hours	• At the tested concentrations, ZEN did not alter measures of monolayer barrier function (notably, electrical resistance) compared to other toxins<br>• ZEN had little effect on expression of tight junction genes at non-cytotoxic concentrations<br>• ZEN increased secretion of the IL-6 cytokine
**In Vivo Studies**					
**Mouse**					
Wang et al., 2013	ICR	ZER	1 to 100 mg/kg (PO)	• N = N/A<br>• Age: 6-8 week old<br>• Treatment Duration: GD 13.5-16.5	• At 100 mg/kg, ZER increased mRNA levels of the P_4 and CRH receptors<br>• Within the placenta, ZER reduced expression of cell cycle proteins and the ERK signaling pathway
Li et al., 2019	C57BL/6/129	ZEN	0.8 to 40 ppm (PO)	• N = 6-9<br>• Age: 2-3 month old<br>• Treatment Duration: GD 5.5-13.5	• At a high concentration (40 ppm), ZEN reduced weights of dams, placentas, and fetuses and reduced the area of placental layers<br>• At a high concentration (40 ppm), ZEN increased placental hemorrhage and resorptions<br>• At all concentrations tested, ZEN increased oil red staining in the labyrinth layer suggesting accumulation of lipids
**Rat**					
Gao et al., 2017	Sprague-Dawley	ZEN	5 to 20 mg/kg	• N=16<br>• Age: NR<br>• Treatment Duration: GD 0-21	• Lower *ERα* mRNA, GnRH receptor, and ATP binding cassette transporters b1 and c1 with 10 and 20 mg/kg ZEN, but higher Abcc5 with 20 mg/kg
Pan et al., 2020	Sprague-Dawley	ZEN	2.5 to 20 mg/kg	• N = 6<br>• Age: NR<br>• Treatment Duration: GD 14-21	• ZEN decreased placenta weight at 20 mg/kg<br>• ZEN decreases placental thickness at 10 and 20 mg/kg<br>• ZEN changes expression of nutrient transporters (*Slc38a1, Echs1, Pc, Slc1a5, Cd36, Ldlr, Hadhb, Cyp17a1*) and Notch signal (*Dvl1 and Jag 1*) measured by qPCR<br>• ZEN reduced phosphorylation of AKT1, ERK1/2, mTOR, 4EBP1<br>• ZEN increased BECLIN1, LC3, p62,<br>• ZEN elevated BAX/BCL2, and CASP3/PROCASP3 ratios

Abbreviations: α-ZOL: alpha-zearalenol; E_2: estradiol; FSH; follicle stimulating hormone; GD: gestation day; IG: intragastric; IM: intramuscular; IP: intraperitoneal; LH: luteinizing hormone; NR: Not reported; PND: post-natal day; PO: per os; P_4: progesterone; PRO: prolactin; SQ: subcutaneous; T: testosterone; ZEN: zearalenone.

Table S7. Impact of Mycoestrogens on Fertilization, Pregnancy, and Fetal Development in vitro and in vivo.

Author, Year	Species, Strain, Cell Type	Compounds Studied	Dose (route)	Timing of Exposure	Main Findings
**In Vitro Studies**					
**Mouse**					
Cao et al., 2019	mESC	ZEN	2 to 20 µg/ml	24 hours	• Strong dose-dependent embryotoxicity
**Pig**					
Alm et al., 2002	In vivo developed gilt zygotes	α-ZOL	3.75 to 30 µM	5 days	• Reduced proportion of zygotes developing to blastocysts and fewer nuclei in those blastocysts starting at 15µM α-ZOL
Malekinejad et al., 2007	COCs	ZEN, α- and β-ZOL	0.312 to 31.2 µmol/L	44 hours	• 12% of treated oocytes formed a blastocyst vs 25% in controls<br>• More aneuploid blastomeres in embryos from exposed oocytes; similar results across metabolites with effects at all doses
Sambuu et al., 2011	Porcine oocytes	ZEN	1 to 1000 µg/L	During fertilization	• No impact on sperm penetration, but at 1000µg/L, positive impact on fertilization rates
Wang et al., 2012	Fertilized porcine embryos	α-ZOL	3 to 60 µM	24 to 84 hours post-insemination	• Decreased cleavage rate starting at 10 µM (dose-response)<br>• Decreased blastocyst development starting at 30 µM<br>• Total cell nos. in blastocysts lower at 10 µM
Yao et al., 2020	Porcine embryos	ZEN	0.5 to 50 µM	24 hours	• ZEN exposure inhibited blastocyst formation at 5 and 50 µM<br>• ZEN exposure increased ROS measured by TUNEL staining and autophagy measured by LC3 and Beclin1
Xu et al., 2020	Porcine embryos	ZEN	10 µM	144 hours	• ZEN decreased 2-cell and blastocyst developmental rate in porcine early embryos<br>• ZEN increased ROS<br>• ZEN increased γH2AX indicating DNA damage, autophagy followed
**Cow**					
Nazar et al., 2017	Bovine oocytes	α- and β-ZOL	3 and 30 µM	During in-vitro maturation	• Decreasing embryo cleavage and blastocyst formation following exposure to α- and β-ZOL, particularly at 30 µM<br>• Reduced cell numbers in 30 µM β-ZOL group (but no other group)
**Human**					

Cao et al., 2019	hESC	ZEN	2 to 20 µg/ml	24 hours	• Strong dose-dependent embryotoxicity<br>• Increased ROS and loss of MMP at 2 and 4 µg/ml ZEN<br>• Induction of cell cycle arrest and apoptosis; upregulation of p53, caspase-9, caspase-3 and ratio of Bax/Bcl-2 at 2 and 4 µg/ml ZEN
**In Vivo Studies**					
**Mouse**					
Wang et al., 2013	ICR	ZER	1 to 100 mg/kg	• N = 8<br>• Age 8 week old<br>• Treatment Duration: GD 13.5 – 16.5	• ZER increased rates of fetal resorption at late gestation(E17.5) and preterm birth<br>• ZER reduced gestational weight gain
Zhao et al., 2013	C57BL/6J	ZEN	0.002 to 40 ppm (PO)	• N = 6<br>• Age: 3/8 week old<br>• Treatment Duration:<br>• Up to 5 weeks	• ZEN reduced percentage plugged mice with implantation sites (9/12 in control vs 1/11 in 40 ppm)<br>• Pregnancy rate 20% in ZEN group compared to 85% in control
Kunishige et al., 2017	Slc:ICR	ZEN	2 to 8 mg/kg (SQ)	• N = N/A<br>• Age: 8 week old<br>• Treatment Duration: GD 1-5	• ZEN reduced the proportion of mice with live fetuses and number of live fetuses/mouse at all doses<br>• Reduced weight of live fetuses at all doses, but no change in placental weight<br>• Fewer implantation rates, with no implantation at highest doses<br>• Inhibition of decidual response following high dose (8 mg/kg) exposure<br>• Inhibition of embryo migration through oviducts causing delayed implantation
Althali et al., 2019	Albino	ZEN	25 mg/kg (PO)	• N = 10<br>• Age: 10-12 week old<br>• Treatment Duration: GD 6-13	• Decreased maternal weight gain in 2nd and 3rd trimesters in ZEN group<br>• ZEN group had significantly more resorbed and dead fetuses, lower fetal body weight and length, more hematoma fetuses and skeletal abnormalities
**Rat**					
Collins et al., 2006	Sprague-Dawley	ZEN	1 to 8 mg/kg (PO)	• N = 27<br>• Age: N/A	• Reduced number of pregnancies at highest dose (81.5% vs 100% in controls)<br>• Reduced maternal weight gain (at 2, 4, and 8 mg/kg) and gravid uterine weight (at 4 and 8 mg/kg)

				• Treatment Duration: GD 6-19	• 8 mg/kg group had more fetal deaths (early and late), more litters resorbed, and more dams with 1+ and 2+ resorptions<br>• Reduced fetal body weight crown-rump length at all doses in both sexes<br>• Skeletal ossification retarded at 4 and 8 mg/kg; more skeletal anomalies at 2, 4, 8 mg/kg
Zhang et al., 2014	Sprague-Dawley	ZEN	0.3 to 146 mg/kg (PO)	• N = N/A<br>• Age: 9 week old<br>• Treatment Duration: GD 0-7	• At higher doses (9, 13.5 mg/kg), reduced maternal weight gain, reduced number of implanted and viable fetuses per litter, more early and late fetal deaths,<br>• At higher doses (9, 13.5 mg/kg), reduced fetal body weight, crown-rump length and brain weight
Gao et al., 2017	Sprague-Dawley	ZEN	5 to 20 mg/kg (PO)	• N = 16<br>• Age = NR<br>• Treatment Duration: GD 0 to 20	• Reduced maternal weight gain<br>• Decrease birth weight and viability at 20 mg/kg
**Pig**					
Trout et al., 2007	Crossbred Gilts	ZER	36 mg (ID)	• N = 40<br>• Age: 5.5 month old<br>• Treatment Duration: 58 days	• ZER group had fewer fetuses, as well as reduced fetal weight, length, and survival
Wu L et al., 2020	Landrace x Large White gilts	ZEN	1 to 10 mg/kg	• N=5<br>• Age: NR<br>• Treatment Duration: GD 7 to 14	• ZEN treatment resulted in smaller embryos<br>• Thicker epithelial cell layer in uterus<br>• ZEN increased autophagosomes<br>• ZEN increased cell apoptosis of primary endometrial cells through Caspase-3 pathway
**Cow**					
Devine et al., 2016	Charolais x Balancer	ZER	36 mg (implant)	• N = 17<br>• Age: 8 month old	• Pregnancy rates (both overall and following artificial insemination) were non-significantly lower in ZER group compared to controls

				• Treatment Duration: 195 days	

Abbreviations: α-ZOL: alpha-zearalenol; E_2: estradiol; FSH; follicle stimulating hormone; GD: gestation day; IG: intragastric; IM: intramuscular; IP: intraperitoneal; LH: luteinizing hormone; NR: Not reported; PND: post-natal day; PO: per os; P_4: progesterone**;** PRO: prolactin; SQ: subcutaneous; T: testosterone; ZEN: zearalenone.

Table S8. PRISMA Checklist.

PRISMA 2009 Checklist

Section/topic	#	Checklist item	Reported on page #
**TITLE**			
Title	1	Identify the report as a systematic review, meta-analysis, or both.	1
**ABSTRACT**			
Structured summary	2	Provide a structured summary including, as applicable: background; objectives; data sources; study eligibility criteria, participants, and interventions; study appraisal and synthesis methods; results; limitations; conclusions and implications of key findings; systematic review registration number.	1
**INTRODUCTION**			
Rationale	3	Describe the rationale for the review in the context of what is already known.	1
Objectives	4	Provide an explicit statement of questions being addressed with reference to participants, interventions, comparisons, outcomes, and study design (PICOS).	6
**METHODS**			
Protocol and registration	5	Indicate if a review protocol exists, if and where it can be accessed (e.g., Web address), and, if available, provide registration information including registration number.	24
Eligibility criteria	6	Specify study characteristics (e.g., PICOS, length of follow-up) and report characteristics (e.g., years considered, language, publication status) used as criteria for eligibility, giving rationale.	24
Information sources	7	Describe all information sources (e.g., databases with dates of coverage, contact with study authors to identify additional studies) in the search and date last searched.	2<br>4
Search	8	Present full electronic search strategy for at least one database, including any limits used, such that it could be repeated.	ST1
Study selection	9	State the process for selecting studies (i.e., screening, eligibility, included in systematic review, and, if applicable, included in the meta-analysis).	26
Data collection process	10	Describe method of data extraction from reports (e.g., piloted forms, independently, in duplicate) and any processes for obtaining and confirming data from investigators.	26
Data items	11	List and define all variables for which data were sought (e.g., PICOS, funding sources) and any assumptions and simplifications made.	26
Risk of bias in individual studies	12	Describe methods used for assessing risk of bias of individual studies (including specification of whether this was done at the study or outcome level), and how this information is to be used in any data synthesis.	26
Summary measures	13	State the principal summary measures (e.g., risk ratio, difference in means).	26
Synthesis of results	14	Describe the methods of handling data and combining results of studies, if done, including measures of consistency (e.g., I^2) for each meta-analysis.	26

Page 1 of 2

PRISMA 2009 Checklist

Section/topic	#	Checklist item	Reported on page #
Risk of bias across studies	15	Specify any assessment of risk of bias that may affect the cumulative evidence (e.g., publication bias, selective reporting within studies).	
Additional analyses	16	Describe methods of additional analyses (e.g., sensitivity or subgroup analyses, meta-regression), if done, indicating which were pre-specified.	
**RESULTS**			
Study selection	17	Give numbers of studies screened, assessed for eligibility, and included in the review, with reasons for exclusions at each stage, ideally with a flow diagram.	5
Study characteristics	18	For each study, present characteristics for which data were extracted (e.g., study size, PICOS, follow-up period) and provide the citations.	Text
Risk of bias within studies	19	Present data on risk of bias of each study and, if available, any outcome level assessment (see item 12).	5
Results of individual studies	20	For all outcomes considered (benefits or harms), present, for each study: (a) simple summary data for each intervention group (b) effect estimates and confidence intervals, ideally with a forest plot.	5-24
Synthesis of results	21	Present results of each meta-analysis done, including confidence intervals and measures of consistency.	
Risk of bias across studies	22	Present results of any assessment of risk of bias across studies (see Item 15).	
Additional analysis	23	Give results of additional analyses, if done (e.g., sensitivity or subgroup analyses, meta-regression [see Item 16]).	
**DISCUSSION**			
Summary of evidence	24	Summarize the main findings including the strength of evidence for each main outcome; consider their relevance to key groups (e.g., healthcare providers, users, and policy makers).	22
Limitations	25	Discuss limitations at study and outcome level (e.g., risk of bias), and at review-level (e.g., incomplete retrieval of identified research, reporting bias).	23
Conclusions	26	Provide a general interpretation of the results in the context of other evidence, and implications for future research.	24
**FUNDING**			
Funding	27	Describe sources of funding for the systematic review and other support (e.g., supply of data); role of funders for the systematic review.	27

From: Moher D, Liberati A, Tetzlaff J, Altman DG, The PRISMA Group (2009). Preferred Reporting Items for Systematic Reviews and Meta-Analyses: The PRISMA Statement. PLoS Med 6(7): e1000097. doi:10.1371/journal.pmed1000097

For more information, visit: **www.prisma-statement.org**.

Page 2 of 2
